# Assessing and accounting for measurement in intensive longitudinal studies: current practices, considerations, and avenues for improvement

**DOI:** 10.1007/s11136-024-03678-0

**Published:** 2024-06-13

**Authors:** Leonie V. D. E. Vogelsmeier, Joran Jongerling, Esther Maassen

**Affiliations:** Department of Methodology and Statistics, Tilburg University, PO Box 90153, 5000 LE Tilburg, The Netherlands

**Keywords:** Experience sampling methodology, Measurement, Reliability, Invariance

## Abstract

**Purpose:**

Intensive longitudinal studies, in which participants complete questionnaires multiple times a day over an extended period, are increasingly popular in the social sciences in general and quality-of-life research in particular. The intensive longitudinal methods allow for studying the dynamics of constructs (e.g., how much patient-reported outcomes vary across time). These methods promise higher ecological validity and lower recall bias than traditional methods that question participants only once, since the high frequency means that participants complete questionnaires in their everyday lives and do not have to retrospectively report about a large time interval. However, to ensure the validity of the results obtained from analyzing the intensive longitudinal data (ILD), greater awareness and understanding of appropriate measurement practices are needed.

**Method:**

We surveyed 42 researchers experienced with ILD regarding their measurement practices and reasons for suboptimal practices.

**Results:**

Results showed that researchers typically do not use measures validated specifically for ILD. Participants assessing the psychometric properties and invariance of measures in their current studies was even less common, as was accounting for these properties when analyzing dynamics. This was mainly because participants did not have the necessary knowledge to conduct these assessments or were unaware of their importance for drawing valid inferences. Open science practices, in contrast, appear reasonably well ingrained in ILD studies.

**Conclusion:**

Measurement practices in ILD still need improvement in some key areas; we provide recommendations in order to create a solid foundation for measuring and analyzing psychological constructs.

**Supplementary Information:**

The online version contains supplementary material available at 10.1007/s11136-024-03678-0.

## Plain English Summary

Collecting patient-reported outcomes (PROs) like depression several times per day over several days or weeks can give valuable insights into how patients are doing in their everyday lives and how much their experiences fluctuate from one moment to the next. These insights have many important (clinical) applications. However, the measurements—the questionnaires to repeatedly assess PROs—need to be of sufficient quality, and if they are not, researchers and practitioners should take that into account. Nevertheless, various aspects of measurement quality often remain uninvestigated, and we do not know the reasons for that. In this study, we identified which aspects are the most commonly omitted and why that is the case. Drawing from these insights, we provide recommendations for good practices that can help establish a solid base for the repeated measurement of PROs over time.

## Introduction

Intensive longitudinal data (ILD) have become increasingly popular for studying psychological constructs as they fluctuate over time. These data are typically collected using experience sampling methodology (ESM; [37]) or related methodologies, in which subjects repeatedly rate questionnaire items measuring psychological constructs several times a day over a prolonged period of time. A key benefit of ILD is that they enable researchers to tap into both within-person fluctuations in psychological constructs and between-person differences in these dynamics. This can be achieved either in isolation or in conjunction with more long-term systematic changes that can also be studied with panel data. In addition, the methods promise higher ecological validity and less recall bias than traditional methods that measure participants only once (e.g., [35]).

Quality-of-life (QOL) researchers increasingly recognize that ILD provide insights into disorders that traditional methodologies cannot provide [5, 35]. For example, ILD can pinpoint when change happens by tracking patient-reported outcomes (PROs) such as well-being or depression in daily life [38, 41], evaluate intervention effectiveness, and offer real-time feedback [4].

However, to reap these benefits of ILD, the repeated measurements need to be of sufficient quality. After all, before drawing conclusions, we need to ensure that what we measure is accurate (i.e., represents the construct we intended to measure) and stable in meaning over time (e.g., ensuring that there are no response shifts, which are not rare in PROs repeatedly assessed using ESM or related methodologies; [25]. Sufficient quality is especially important because of the high clinical relevance in health research [4, 41]. Nevertheless, how to operationalize (psychological) constructs (i.e., how to quantify and use them as PROs) and how to assess and account for psychometric properties of these instruments in analyzing dynamics remains challenging for researchers working with ILD.

Researchers may choose suboptimal measurement practices and considerations for a variety of reasons. Some may lack suitable methods for evaluating items or scales, while others may lack awareness, skills, or software to use existing methods. This study aims to shed light on the reasons behind researchers’ measurement choices. In the following, we summarize ways to operationalize psychological constructs and what measurement practices and considerations are generally important for ILD studies and highlight the current shortcomings before justifying the survey in this study.

## Operationalizing psychological constructs

Several procedures for instrument selection are currently used in ILD studies, ranging from creating new items to adapting or rewording questionnaires that have been used in previous studies. Possible strategies to obtain scale scores from the selected items include single-item scores, sum-, average- or maximum scores of multiple items, and factor or component scores (for a discussion of some of these approaches, see [6]). The selection and scoring of items will generally depend on the research question. For instance, researchers who want to study related constructs like positive and negative affect separately should construct two separate scores, while researchers interested in controlling for the overlap between affect and other constructs may choose a single continuous affect item (for an extensive discussion on this, see [6]).

As Flake and Fried [11] pointed out, there is likely no single psychological construct for which there is only one validated measurement universally accepted by the field, without any degrees of freedom regarding how to obtain a construct score. Because of this, it is crucial for researchers to carefully assess and account for the psychometric properties of the chosen instruments.

## Assessing and accounting for psychometric properties

Regardless of the chosen type of scale, researchers should assess and account for the psychometric properties of instruments, including reliability, factor structure, and measurement invariance across subjects and time to ensure the validity of subsequent analyses (e.g., [7, 28, 45]). For example, sum scores assume that each item measures the construct equally well [28, 29], which may not be realistic and needs to be investigated by a factor analytic evaluation of the items. It is important to assess the properties even if the instrument has previously been evaluated, as instruments that perform well in cross-sectional data have limited applicability in ILD [10, 13, 14, 44]. Furthermore, instruments for ILD that work well in one sample or context do not have to work well in another sample or context [6]. Moreover, from cross-sectional research, we know that various scales in psychology are unsystematically developed and adapted [12, 46] and show poor validity evidence regarding the factor structure and measurement invariance [15, 24]. These problems may be further compounded in ILD studies, where consensus has not even been reached yet on how to measure common constructs [31].

After the psychometric evaluations, the outcomes need to be included in the analyses. For instance, if multiple items are used to assess a construct and the assumptions of sum scores do not hold in the data, the adequate factor structure and, if required, (partial) non-invariance must be considered in subsequent analyses (for example, using dynamic structural equation modeling; DSEM; [1, 22]). Failing to do so may lead to biased parameter estimates [8, 9, 21, 28, 32]. Note that it is assumed that the factor model correctly describes the data generating mechanism, highlighting the importance of careful psychometric evaluation.^1^

The need and benefit for proper assessment and accounting for measurement properties in ILD studies has been extensively discussed in the psychometric literature (for an overview, see [28]) but is not common practice yet. Additionally, current reporting practices in research lack sufficient detail on measurement (e.g., [12, 15, 24]), which prevents researchers from fully evaluating the validity or robustness of results [16, 34], and limits them from confidently building upon a study’s conclusions in subsequent research [11]. Furthermore, this limited attention to measurement makes it hard to gain insight into researchers’ methodological choices and the reasons behind them through investigating the literature. To address these issues, we opted for a survey-based approach in this study since the required information would not be well captured in a meta-study.

## Current study

This survey evaluates the measurement practices of academic researchers working with ILD, as well as the reasons behind any suboptimal practices. This is a crucial first step in (i) raising awareness of the importance of appropriate measurement practices and providing transparent motivations, (ii) identifying which measurement topics need further research and development, and (iii) assessing what type of resources and educational materials would benefit researchers in their analyses. We identify the areas in most need of improvement and provide recommendations for applied researchers and other methodologists, drawing on the latest technical and methodological developments in the field of ILD.

## A survey to shed light on measurement practices and considerations

We conducted our survey using the online platform Qualtrics. To ensure the clarity of the survey questions, we first sent the final draft to two ILD experts and a psychometrician who were not part of the subsequent sample. Based on their feedback, we made necessary adjustments to the survey. The final survey took approximately 8 min to complete. The Ethics Review Board of Tilburg University approved the study under project number TSB_RP558. The data collection period started in September 2022 and ended on December 31, 2022.

## Procedure and materials

We invited all members from the Dutch-Belgian ESM network to participate in the survey through e-mail, the Basecamp platform, Twitter, and Mastodon, and we advertised the survey during the ESM network meeting in September 2022 in Leuven. While geographically limited, members of the Dutch-Belgian ESM network make up a large proportion of researchers using ESM or related methodologies worldwide. The aim of the study was communicated in the invitation letter and in the informed consent section at the beginning of the survey. After agreeing to participate, participants answered a series of questions, starting with questions about their expertise and background. Subsequently, they answered a series of questions regarding the most recent article for which they were the first author and which involved ILD analysis. Specifically, the participants answered questions about their study, measurement practices and considerations, analyses conducted, open science practices, and confidence in their analyses. The survey questions and a concise summary of the survey content are available in Supplemental Material B.

## Results

### Expertise and background

Our sample consisted of 42 participants after removing one participant who indicated they had not designed or analyzed any ILD studies and one participant with implausible answers (i.e., reporting the analysis of over 300 constructs and using numbers instead of names for the construct). Although small, this sample represents over one-third of our target population (i.e., the Dutch-Belgian ESM Network). Therefore, this sample should give a reasonable insight into the field. A complete overview of the results of this survey is available in Supplemental Material C. Most participants indicated they were moderately, very, or extremely knowledgeable on the topic of measurement of psychological constructs in general (*N* = 37), as well as measurement of psychological constructs in ILD studies in particular (*N* = 35). Only a small number of participants (*N* = 5 for general studies, *N* = 7 for ILD studies) reported having little to no knowledge of these topics. The number of ILD studies (co-)designed ranged from 0 to 40 (mean = 3.3, median = 2). The number of ILD studies (co-)analyzed ranged from 1 to 40 (mean = 5.4, median = 4).

The most frequently reported background field was Methodology and Statistics (*N* = 14), followed by Clinical Psychology (*N* = 12) and Developmental Psychology (*N* = 7). Of the 28 participants with no background in Methodology and Statistics, 3 indicated that they collaborated with a methodologist, statistician, or psychometrician to analyze the data.

## Study information

The studies referenced by participants varied considerably in sample and measurement characteristics. Specifically, the number of subjects participants used for the analyses ranged from 1 to 10,000 (mean = 666, median = 90), the number of measurement occasions ranged from 3 to 4037 (mean = 321, median = 70), and the number of psychological constructs ranged from 1 to 15 (mean = 4.3, median = 3).

## Measurement practices and considerations

### Construct measurement

The participants in our study were asked to answer questions on a maximum of two constructs they had previously analyzed. Thirty-seven participants responded to questions on 62 constructs.^2^ Among these constructs, 29 were measured using a single item, and 26 constructs were assessed using multiple items, with the number of items ranging from 1 to 15 (mean = 2.6, median = 1). For the remaining seven constructs, the number of items was not specified by the participants. Please note that the remainder of this results section does not use the participants, but the number of constructs as the unit of analysis.

### Scale validation

For 14 of 26 constructs measured with multiple items, adequate attention was paid to measurement practices, as they had been evaluated for reliability and factor structure in a previous or the current ILD study.^3^ Specifically, five were evaluated both in a previous ILD study and in the current one^4^, six were evaluated only in a previous and not the current ILD study^5^, and three were evaluated only in the current and not in a previous ILD study. In contrast, 12 constructs did not undergo appropriate evaluation as they were neither evaluated in a previous nor the current ILD study.

The reasons participants provided for not evaluating the reliability and/or factor structure of a construct were primarily that they did not know that this was relevant for drawing valid inferences in their analyses (seven times), or they did not know how to do this (six times). Other reasons include not having enough items to run the factor analysis, assuming that Cronbach’s alpha is sufficient to establish the factor structure, deeming the validation process unnecessary for their study (such as when the analysis was solely used as an illustrative example), and recognizing the importance of assessing the factor structure, but deciding against doing so.

### Item validation

For 12 of 29 constructs measured using a single item, appropriate measurement practices were followed, as they had been evaluated for reliability in a previous or the current ILD study.^6^ Specifically, two items were evaluated in both a previous and the current ILD study, six were evaluated only in a previous and not the current ILD study^7^, and four were evaluated only in the current and not in a previous ILD study. In contrast, 17 items did not undergo appropriate evaluation as they were neither evaluated in a previous nor the current ILD study.

The most common reasons for not evaluating item reliability in their current study were that participants did not know how to evaluate reliability (10 times) or they did not know it may be relevant for drawing valid inferences (four times). Some participants thought that reliability analysis was unnecessary, either because they only used one item, the measure was very explicit, previous studies had assessed reliability, or it was not relevant to the study's goal. Sometimes, reliability analysis seemed not feasible or appropriate given the participant’s data. Lastly, one participant indicated they did not include reliability analyses in their manuscripts because reviewers and editors would request it to be removed from the manuscript to preserve space.

### Measurement error and invariance

If a construct was measured by one item or multiple items were averaged or summed, participants were asked if they corrected the construct score for measurement error. Participants reported doing so for only three out of 51 constructs. Participants using constructs measured by multiple items were asked if they assessed some type(s) of measurement invariance in their model. Out of 26 constructs measured by multiple items, four were assessed for measurement invariance, 21 were not, and for one construct, the participants reported they did not know or remember. We asked participants for the reasons they did not assess measurement invariance for their construct. Participants could indicate that they did not know how (indicated nine times) or that they did not know it was relevant for drawing valid inferences (six times). Additionally, participants could formulate their own reasons. Other explanations included the perceived irrelevance of measurement invariance testing for the type or topic of the study (seven times), models being already too complex (two times), an excess of other analyses in the study (two times), a conscious decision not to test for it (two times), or time constraints (indicated once).

For the four constructs that were assessed for measurement invariance, participants indicated that none reached full measurement invariance [here defined as *(partial) residual invariance*]. For two constructs, follow-up steps were taken to correct for measurement non-invariance; for one, random effects on item parameters were used, whereas for the other construct, the follow-up steps were not further clarified. For the two other constructs, no follow-up steps were taken to correct for measurement non-invariance because the participant did not know it may be relevant for drawing valid inferences for their analyses.

### Levels of invariance

Different levels of measurement invariance are required for various types of analyses. For example, analyses that focus on dynamics (e.g., correlations) require (partial) loading invariance, while mean differences between groups require (partial) intercept invariance. Table 1 provides an overview of how often different types of invariance were reported to be tested, which minimum level of invariance is required, and how often the required level of invariance was reported to be achieved for six possible types of analyses.

**Table 1:** Information on the invariance for each type of analysis conducted

Type of analysis	Number of constructs for which the analysis in column 1 was applied	Required level of (partial) measurement invariance	Reported invariance assessment	Number of constructs with verified appropriate level of invariance
Differences between independent groups (e.g., patients vs. non-patients) in the dynamics of the construct	9	Longitudinal and between-group loading invariance	2× between-person invariance, invariance across subject- and/or time-specific covariates/groups, invariance across within- and between-person level 2× between-person and longitudinal invariance	1
Mean differences in the construct across independent groups (e.g., patients vs. non-patients)	7	Longitudinal and between-group loading- and intercept invariance	2× between-person invariance, invariance across subject- and/or time-specific covariates/groups, invariance across within- and between-person level 1× between-person and longitudinal invariance	0
Differences between dependent groups (e.g., before vs. after intervention) in the dynamics of the construct	3	Longitudinal and between-group loading invariance	1× between-person and longitudinal invariance	0
Mean differences in the construct across dependent groups (e.g., before vs. after intervention)	3	Longitudinal and between-group loading- and intercept invariance.	1× between-person and longitudinal invariance	0
Relationship(s) between the construct and other constructs (e.g., the correlation between depression and anxiety or depression and itself)	43	Longitudinal and between-group loading invariance	2× between-person invariance, invariance across subject- and/or time-specific covariates/groups, invariance across within- and between-person level 2× between-person and longitudinal invariance	1
Time trend in the construct	32	Longitudinal and between-group loading- and intercept invariance	2× between-person invariance, invariance across subject- and/or time-specific covariates/groups, invariance across within- and between-person level 2× between-person and longitudinal invariance	1

### Analyses conducted

The four most common analyses were assessing relationship(s) between the construct and other constructs (43 times), time trends (32 times), differences between independent groups in the dynamics of the construct (nine times), and mean differences in the construct across independent groups (seven times). Analyzing differences between dependent groups in the dynamics of the construct and analyzing mean differences in the construct across dependent groups were not common (both three times). The top three types of analysis used were Multilevel Regression (13 times for single-item and 12 times for multi-item measures), Multilevel (V)AR modeling (six times for single-item and five times for multi-item measures), and Dynamic Structural Equation Modeling or Dynamic Factor Analysis (seven times for single-item and two times for multi-item measures).

### Open science practices and confidence

For almost all 62 constructs, participants stated that the analysis steps were reported in such detail that the reader could reproduce the analyses if they had the data (50 times). The syntax or code for all the analysis steps was stated to be publicly available for about half of the constructs (33 times). Most participants were moderately confident that the analyses were ideal for their research design (25 times) or slightly confident (11 times). A complete list of results for the study is displayed in Supplemental Material C.

## Discussion

An increasing number of researchers are using ILD to study dynamics in psychological constructs in social sciences in general and in QOL research in particular, where this method is applied to PROs. However, before we can reap the benefits of ILD, we need a solid foundation of ILD measurement and an understanding of factors contributing to suboptimal practices. Drawing on our survey results, this study pinpoints researchers’ most common measurement considerations and practices when working with ILD and indicates why researchers may choose suboptimal practices. Our objectives were to raise awareness of the importance of appropriate measurement practices and transparent motivations, identify measurement topics that require further research and development, and determine the types of resources and educational materials that would aid researchers most. Below, we briefly summarize the key findings and provide recommendations for researchers working with ILD. We end with discussing future directions for the broader academic context, including reviewers, editors, and funding agencies.

## Summary

Results showed that a methodologist, statistician, or psychometrician was involved in almost half of the data analyses. In addition, participants were positive about the suitability of their analyses and their ability to make appropriate measurement choices; almost all participants were (1) at least moderately confident that their analyses were ideal for their research design (including the assessment of measurement invariance and the evaluation of psychometric properties), and (2) rated their knowledge about measurement in general and in ILD studies in particular as at least moderate. Most analyses were conducted using either single-item measures or average scores of multiple items. The psychometric properties of these measures were only evaluated specifically for ILD in fewer than half of the multi-item measures and in approximately one-third of the single-item measures. Assessment of psychometric properties in the participants’ current studies was even less common—mainly because participants did not have the necessary knowledge to conduct these assessments or were unaware of their importance for drawing valid inferences. Although some attention was given to psychometric properties of the instruments, they were often not taken into account in the analyses of ILD studies. For example, for multi-item instruments, multilevel (V)AR models (treating construct scores as observed) were more frequently used than dynamic structural equation models (that treat scores as latent by taking measurement models into account).

The most significant issue identified in ILD studies was the rare assessment of measurement invariance, and that even when it was assessed, the required level of invariance for the research question was usually not achieved. The most common reasons for not investigating measurement invariance, similar to those for psychometric properties, included not knowing how to conduct the assessment, as well as not recognizing or underestimating its significance in drawing valid inferences.

Finally, open science practices appeared reasonably well ingrained in ILD studies as most participants indicated that they reported their results in such a way that they would be reproducible, and half of the participants indicated they shared the syntax and code for all the analysis steps publicly online.

Our following recommendations pay particular attention to the gaps identified in the results of the survey. However, it is important to note that the results of the survey are constrained regarding sample size and to exercise caution when interpreting and generalizing the results. A survey with different wording and (orders of) response options might have led to slightly different results. For example, for all questions asking about the reasons why a researcher did not evaluate psychometric properties, the options “I did not know it was relevant[…]” and “I did not know how” were the first two response options and thus possibly chosen the most partly due to the ease of selecting them (e.g., [20]). Nevertheless, these survey results should be regarded as initial insights into measurement considerations in ILD studies, which researchers can build upon in future studies (e.g., Delphi studies, e.g., see [40]).

## Recommendations

To date, only few studies have focused on assessing and accounting for measurement (and thus the psychometric properties) in intensive longitudinal studies. In the following, we combined insights from these studies with the findings of this survey to provide some recommendations for good measurement practices in ILD. It is important to note that intensive longitudinal measurement is a young and evolving field, and best practices will likely change or be updated over time. As such, the following recommendations (on single-item measures, multi-item measures, and measurement reporting and sharing; Box 1, Box 2, Box 3) should be viewed as indicators of current good practices.

Box 1: Recommendations for single-item measures

- Check if there are already single-item measures for your construct whose reliability has been assessed in ILD with similar characteristics to your data (with respect to study population, sampling design, etc.), and use those if available (e.g., using the ESM item repository; [19])
- Assess the reliability of each item in your study (even if you use items for which the reliability had been previously established) by incorporating a test-retest procedure in your design (e.g., randomly repeat emotion items within the same questionnaire; [7]). Assessing reliability with methods other than this test-retest approach is challenging, as the item scores are expected to change over time in ILD studies. Note, however, that while memory effects may artificially decrease measurement error, the participant’s annoyance, reactivity, or confusion upon encountering the same item in quick succession may elevate measurement error [7]. An alternative approach for indirectly evaluating the reliability for single items in autoregressive models involves running the measurement error (V)AR model [36] and calculating the reliability by dividing the true variance by the error variance (also referred to as the signal-to-noise ratio, [7]). However, this method may face convergence problems if the autocorrelation is low [36]. Note that example code for conducting the measurement error VAR model is provided along with Supplemental Material A.
- Choose an analysis where you can account for reliabilities. Otherwise, you run the risk of drawing incorrect conclusions about the dynamics of psychological constructs. You may choose a model where reliability is automatically assessed and accounted for (e.g., for autoregressive analyses, you may look into the measurement error (V)AR model [36]). If you can assess reliability by design, for example, using the test-retest approach by Dejonckheere et al. [7], you may account for reliability manually with the measurement error (V)AR model (by adding constraints to measurement error term based on the reliability of that item). This last approach would also aid with the convergence issues that can arise with this model in case of low autocorrelation [36]. Note that reliability is a complex topic as opinions about what the systematic- and total variances that should be used differ (especially when using multilevel models, [22]). Researchers should therefore think critically about what variances are appropriate for their goal.
- Pilot your instrument in an independent representative sample if the instruments are new, revised, or used in other contexts [47].

Box 2: Recommendations for multi-item measures

- Check if there are already multi-item measures for your construct whose reliability and factor structure have been assessed in ILD with similar characteristics to your data (with respect to study population, sampling design, etc.), and use those if available (e.g., using the ESM item repository; [19]).
- Evaluate the factor structure of your measure even when you want to use sum scores (or mean scores). Sum scores make a strong assumption about the underlying factor structure, namely that the factor loadings are approximately equal across items and that measurement error is equal and minimal for each item. These assumptions can be checked using the DSEM analysis framework of Mplus [27, 30] or using self-written code for Stan [39]. Both software types allow for fitting dynamic factor models to your data. A Stan code example is provided along with Supplemental Material A. If the assumptions underlying sum scores are violated, which they likely are, the dynamic factor models are the more suitable alternative. Researchers should be aware that DSEM [27] assumes that the dimensionality of the factor structure is invariant across time, which might not be the case (e.g., because of response shifts). This could be problematic because, if the dimensionality of the factor structure indeed changes across time, but this is not taken into account in the analyses, researchers could conflate changes in dimensionality with actual changes in the underlying construct. To investigate across time-invariance in dimensionality, researchers can use latent Markov factor analysis (LMFA) [45].
- Verify if the required level of measurement invariance across subjects/time holds for your research question (see Table 1). You can evaluate gradual differences in parameters by inspecting the variance in random effects in cross-classified factor analysis [28] and assess qualitatively distinct (context-specific) factor structures using LMFA [45]. If either of these methods indicates that measurement is non-invariant across subjects/time, a model with the appropriate random effects for the measurement parameters (using DSEM or Stan) or LMFA models should be considered your baseline model to which you can add further covariates while taking the non-invariance into account. Note, however, that both approaches have limitations. For cross-classified factor analysis, it is unclear how much variance truly signifies invariance violations, as this depends on the sample characteristics [28]. Similarly, LMFA requires a complex model selection procedure to determine how many qualitatively different factor structures underlie the data, which is inherently prone to researchers’ subjective decision-making, even when supplemented with model fit indices like the BIC [42]. In addition, the approaches are complex, requiring experience in model specification. To gain this experience, consider examining empirical applications that provide syntax, model specification, and interpretations of the results (for examples of cross-classified factor analysis and subsequently accounting for (partial) non-invariance, see [18, 28], for examples of LMFA, see [42, 43]).
- Ideally, integrate the factor structure of your measurements into your analyses (e.g., into the models used to estimate the relations between constructs) using DSEM [27, 30] or Stan [39]. Note that extracting factor scores for individuals from a factor model and using these scores in separate follow-up analyses is not straightforward and requires special corrections to prevent bias in the estimates of standard errors [8, 9, 23], which are not tailored to ILD yet.
- If a factor structure cannot be integrated into the analysis (e.g., because that would make the model too complex for the data at hand, which could be indicated by convergence problems), an alternative approach is to use sum scores but to account for measurement error when analyzing any dynamics in the constructs, for example, using the measurement error (V)AR model [36]. An example of this for simulated data is provided in Supplemental Material A. However, be aware that convergence problems are probable in empirical data when the autocorrelation is low [36]. Note that, like DSEM, the measurement error (V)AR model [36] assumes that the dimensionality of the factor structure is invariant across time, which might not be the case (e.g., because of response shifts). Researchers can use LMFA [45] to check for changes in dimensionality.
- Assess the reliability of your construct. For example, you can compute McDonald’s Omega [26] using the estimates of factor loadings and measurement errors obtained from DSEM or Stan, ideally for the within- and between-person level separately [22]. Note, however, that reliability in the context of multilevel data has unique challenges because factor scores (or other composite scores) can be constructed at both levels [17, 22]. One important consideration is that if one wants to interpret between-person-level factors as the means of the within-person factors (i.e., the typical interpretation when applying multilevel analysis to ILD), it is necessary to establish cross-level invariance. This means verifying that the measurement model at the within-level is identical to that at the between-level in terms of dimensionality, factor loadings, and intercepts. For example, suppose a researcher studying positive and negative affect in ILD finds the exact same 2-factor model on both the within- and between-person levels. Then, the two factors on the within-level represent participants' momentary positive- and negative affect, while the two factors on the between-level represent participants' average positive and negative affect across all repeated measurements. If cross-level invariance is violated, the interpretation of the between-person level factors is unclear. In this example, the two factors on the within-level would still represent momentary positive and negative emotion scores, but the between-level factors would not be the person averages of these constructs. Therefore, one may want to refrain from using a factor model to model between-person-level variances when cross-level invariance is violated (and, consequently, from using a factor model to determine reliability at the between-person level). Instead, one could freely estimate (co)variances at the between-person level.
- Pilot your instrument in an independent representative sample if the instruments are new, revised, or used in other contexts [47].

Box 3: Recommendations for measurement reporting and sharing When using single- or multi-item measures of psychological constructs, report:

- A citation to a previous (ILD) study where the item or scale was validated or assessed for reliability in the same or similar population;
- Any modifications made to the item or scale in the current study;
- All psychometric properties of the item or scale (e.g., level of measurement, item response categories);
- All analyses conducted (e.g., correction for measurement error, factor analysis), and decision criteria used (e.g., cut-off values for factor selection decision criteria);
- A reliability estimate (e.g., McDonald's Omega; [26]), for the entire sample and all analyzed groups,
- If applicable, the factor structure of the construct, including model fit statistics;
- If applicable, measurement invariance analyses, including the type of test, order of model fit, achieved level of invariance, and model fit statistics following reporting guidelines. These are, however, currently only tailored to cross-sectional or panel data (e.g., by [34]) and not to ILD. We advise researchers to stay attentive for forthcoming guidelines in this area.

When disseminating the results of your study, share:

- The reliability information from your study (e.g., using the ESM item repository; [19]),
- An anonymized and GDPR-compliant data set, including a codebook with clear variable descriptions and code to re-run all reported analyses (e.g., using OSF; [33]).

## Future directions

Our survey identified key areas where measurement practices in ILD need improvement. Specifically, there is a shortage of empirically validated measurement instruments for ILD, as indicated by the fact that most studies used measures that were not psychometrically evaluated specifically for use with ILD. In addition, there appear to be gaps in knowledge and tools for incorporating psychometric properties in analyses and assessing measurement invariance.

To develop our toolbox of empirically validated ILD measurement instruments, journal editors can request that researchers provide detailed information on how they operationalized their construct and assessed psychometric properties, measurement invariance, and the results thereof. Journals and funding agencies can require researchers to provide such information, similar to how they do for code, syntax, and data. Templates and journal guidelines for providing this information could be developed (for both single-item and multiple-item measures), which would aid reviewers in verifying whether researchers have provided all relevant measurement information. Moreover, it is important that the ILD community conducts research specifically on instrument development in ILD, similar to current practices in cross-sectional research. Editors and journals could facilitate this process by planning special issues focused on these topics.

To fill the knowledge gaps and provide researchers with the necessary tools to incorporate psychometric properties in analyses and test for measurement invariance, these topics should be integrated into graduate training, as well as (online) tutorials and webinars. Investigating psychometric properties and invariance requires complex approaches, and (future) researchers need to be adequately prepared to handle these. Additionally, applied researchers should collaborate with methodologists with expertise in these areas. Editors could ask at least one methodologist with such expertise to be on the review team, thereby raising awareness and sharing current insights on these topics.

In addition to theoretical training, there is a need to simplify the process of applying this knowledge in one’s own research. Currently, only a few statistical software packages allow for accounting for the factor structure when analyzing dynamics in constructs, and no single package allows for the assessment of both psychometric properties and invariance. In the short run, tutorials with easily accessible code and webinars could help address this issue. In the long run, these options would ideally be made available to ILD researchers in a single, freely accessible software package.

Finally, we view improving measurement in ILD as an ongoing shared responsibility and believe that the ILD research community should support each other whenever possible. A great example of such collaborative effort is the ESM item repository [19], which provides researchers with an overview of instruments used in previous ILD studies. Every researcher can contribute by making their new instruments available via this repository. In the future, it would be beneficial to have extended information on psychometric properties and invariance assessment of instruments in varying contexts (e.g., regarding population, study protocol, and situational contexts). These insights would allow researchers to screen the repository for potentially suitable instruments, quickly identifying those that worked well in studies conducted under similar conditions. Greater emphasis on qualitative ILD research is also desirable. For instance, incorporating participant feedback on their understanding and responses to questionnaire items can be valuable in refining and improving the quality of ILD gathering instruments [2, 3, 38]. With every step of creating, improving, assessing, and accounting for measurement, we move closer to forming a solid foundation for measurement in ILD.

### Supplementary Information

Below is the link to the electronic supplementary material.

Supplementary file1 (PDF 142 KB)

Supplementary file2 (PDF 3120 KB)

Supplementary file3 (HTML 1059 KB)

Supplementary file4 (PDF 235 KB)

## Data Availability

The data are not publicly available due to their containing information that could compromise the privacy of research participants.

## Footnotes

1 In Supplemental Material A, we demonstrate how ignoring psychometric properties in analyses can bias results and invalidate conclusions in the context of a multilevel first-order autoregressive (AR(1)) model.

2 The construct questions were unanswered by the remaining five participants.

3 Note that we did not report how many indicated “only reliability” or “only factor analysis”, as both assessments are required for multiple-item scales in ILD studies. Similarly, we omitted the results for evaluation of reliability and factor analysis in previous non-ILD studies because evaluation in previous ILD studies and/or the current ILD study is necessary. However, detailed results can be found in Supplemental Material C.

4 This was particularly important for two of the constructs that underwent modifications (e.g., by adding items).

5 It is important to note, however, that four of the six constructs underwent modifications and should ideally have been assessed in the current study again.

6 Note that we have omitted the results for evaluation of reliability in previous non-ILD studies because evaluation in previous ILD studies and/or the current ILD study is necessary. However, detailed results can be found in Supplemental Material C.

7 It is important to note, however, that two of the six items underwent modifications and should ideally have been assessed in the current study again.
