# Supplementary material for: Assessing and accounting for measurement in intensive longitudinal studies: current practices, considerations, and avenues for improvement
Source: Qual Life Res. 2024 Jun 13;33(8):2107–18. doi: 10.1007/s11136-024-03678-0 (PMC11286633; doi:10.1007/s11136-024-03678-0)
Supplement: Supplementary file 1 — Supplementary file1 (PDF 142 KB) [file 11136_2024_3678_MOESM1_ESM.pdf]

Supplemental Material A

for "*Assessing and Accounting for Measurement in Intensive Longitudinal Studies: Current Practices, Considerations, and Avenues for Improvement*"

To demonstrate the effect of erroneously ignoring the factor structure of your measure on parameters obtained from a multilevel AR(1) model (Jongerling et al., 2015), we generated ILD from a one-factor model in which the underlying latent construct is measured by five observed items and in which there is an AR(1) process between the factor scores. The factor loadings for the five items are equal to .90, .30, .25, .80, and .50. The residual variances of the items are equal to .30. With these values, the variance in the items explained by the factor varies from 17% for item 3 to 73% for item 1. Item intercepts are fixed to 0, and for identification purposes, the mean and variance of the latent construct are fixed to 0 and 1, respectively. In addition, individual AR parameters are sampled from a normal distribution with a mean of .40 (i.e., the fixed effect) and a between-person *SD* of .10.

We generated scores based on this (factor) AR(1) model for a sample size of $N = 100$ and $T = 50$ repeated measures. Thus, we generated scores on all five items 50 times, for 100 different individuals, with scores on each of the 50 occasions following the one-factor measurement model described previously and the individual factor scores following the described AR(1) process.

The generated data were analyzed in two ways. First, we fit a Multilevel Dynamic Factor Model that is in line with the data-generating mechanism. Second, we calculated mean scores across the five items for each timepoint and individual separately, resulting in 50 average scores for each of the 100 subjects (i.e., one for each measurement occasion). These mean scores were subsequently analyzed with a multilevel AR(1) model (hereinafter referred to as Mean Score Multilevel AR(1) Model). Both analyses were compared in terms of bias in the estimated mean AR parameter (i.e., in the fixed effect) and bias in the between-person *SD* of the AR parameter, as these are typically the parameter of central interest with this type of

model. In addition, we compared the mean absolute distance (MAD) and correlation between estimated and true individual AR parameters. We investigated the MAD to see how far off the individual AR estimates are from their true values, while the correlation tells us to what extent the rank order of the individual estimates is preserved (i.e., to what extent individual differences in AR parameters are accurately recovered).

We ran our simulation for 500 replications as initial results showed that this number of replications would ensure that the MCMC-error (i.e., the imprecision in our results resulting from the simulation procedure) would be no larger than .01. This implies that, in our simulation results, only differences between true and estimated parameter values larger than .01 should be considered actual bias in the results of a method, as smaller differences could just be numerical inaccuracy. Results are presented in the first two rows of Table 1. When applying the Mean Score Multilevel AR(1) Model, the underestimation in the overall mean AR parameter (i.e., the fixed effect) is equal to -.056 (14.00%), while bias is only .007 (1.75%) when using the proper Multilevel Dynamic Factor Model. The latter is well below the MCMC error. In addition, the between-person *SD* of the AR parameter is underestimated by .009 (6%) when using the Mean Score Multilevel AR(1) Model. In comparison, the bias in the *SD* is .004 (4%) when using the Multilevel Dynamic Factor Model. For both models, the bias in the *SD* is smaller than the MCMC error, however. For the individual AR parameters, the Mean Score Multilevel AR(1) Model performs worse in terms of both MAD and correlation between estimated and true individual parameters, although the difference in correlations between the two approaches is quite small. Overall, simply using mean scores in a multilevel AR(1) model leads to substantial bias, especially in the overall and individual estimates of the AR parameter. The *SD* estimates of this parameter are also affected, albeit to a lesser degree. Note that the bias could be reduced by using the mean scores with the

measurement error (V)AR model (Schuurman & Hamaker, 2019). The simulation study results including this analysis are displayed in the third row of Table 1.

Table 1

Bias in the Parameter Estimates for Three Modeling Approaches

Model/Parameter	Overall AR-parameter		Individual AR-parameter	
	Bias Fixed Effect	Bias *SD*	Correlation	MAD
Multilevel Dynamic Factor Model	.007	.004	.561	.068
Mean Score Multilevel AR(1) Model	-.056	.009	.551	.082
Measurement-Error AR(1) Model	-.013	.007	.554	.071

Note. The mean absolute distance (MAD) shows how far off the individual AR estimates are from their true values, while the correlation tells us to what extent the rank order of the individual estimates is preserved (i.e., to what extent individual differences in AR parameters are accurately recovered).
