# Supplementary material for: Assessing and accounting for measurement in intensive longitudinal studies: current practices, considerations, and avenues for improvement
Source: Qual Life Res. 2024 Jun 13;33(8):2107–18. doi: 10.1007/s11136-024-03678-0 (PMC11286633; doi:10.1007/s11136-024-03678-0)
Supplement: Supplementary file 2 — Supplementary file2 (PDF 3120 KB) [file 11136_2024_3678_MOESM2_ESM.pdf]

Supplemental Material B

for "*Assessing and Accounting for Measurement in Intensive Longitudinal Studies: Current Practices, Considerations, and Avenues for Improvement*"

We begin with a concise summary of the material and afterwards provide the codebook.

Summary

Expertise and Background

Regarding expertise, participants indicated the number of ILD studies they have designed and analyzed, as well as their knowledge on the topic of measurement of psychological constructs in general and in ILD studies in particular. Additionally, participants indicated which field(s) best described their background and their position when analyzing the data (ranging from junior researcher to full professor).

Study Information

Participants were asked about the status of the article they chose to focus on in the survey and, depending on their answers, indicated the year their article was accepted for publication, submitted for review, or uploaded as preprint. Participants that did not indicate methodology and statistics in their background were asked whether they collaborated with a methodologist, statistician, or psychometrician to analyze the data.

Next, participants were asked to answer questions about the characteristics of the article. In case the article contained multiple studies and/or multiple research questions, participants were asked to select the study and/or research question they deemed most central or interesting and to use this study to answer the subsequent questions. Participants indicated the (approximate) number of subjects they used for the analyses, the (approximate) average number of measurement occasions per subject they used, and how many psychological constructs they analyzed in their chosen study. Finally, the participants were asked to keep their first and second constructs in mind while answering the subsequent questions about measurement practices and considerations.

Measurement Practices & Considerations

Construct measurement

To assess construct operationalization and validation, we asked participants to indicate for each construct how many items were measured and the approach used to obtain construct scores (e.g., the construct scores were average scores across multiple items, the construct scores were based on the scores on a single item, the construct scores were factor scores obtained using a factor analysis model).

Scale/item validation

Participants were asked three questions for which they indicated whether they used a scale or item for which the reliability and/or factor structure were validated (1) in previous ILD studies, (2) in previous research other than ILD studies, and (3) if they validated the reliability and/or factor structure in the current study. If they had not evaluated the reliability and/or factor structure in the current study, participants were asked what the reason was for not doing so. In addition, participants were asked if they modified the scale or item in any way (e.g., by adding, removing, or translating items).

Measurement error and invariance

Next, for constructs with one item or averaged or summed multiple items, participants were asked if they corrected the construct for measurement error. For construct scores that were created through factor analysis, item response theory, or other methods, participants were asked if they assessed some type(s) of measurement invariance, and if yes, what type (e.g., across subjects, across time). For each indicated type of invariance assessment, participants were then asked to indicate what level of invariance held (ranging from no level of invariance held to (partial) residual invariance). If they indicated for any invariance test that they had not reached full invariance (i.e., (partial) residual invariance), participants were asked if they had conducted any follow-up analyses and, if yes, participants could describe in a text box the specific follow-up steps they had taken.

Additionally, if participants did not correct for measurement error, assess measurement invariance or if they did not do any follow-up analyses to correct for measurement non-invariance, they were asked to provide a reason why they had not done so (e.g., I did not know how, I did not know that it may be relevant for drawing valid inferences for my analyses).

Analyses Conducted

For the type of analyses participants conducted, participants were first asked whether they performed any of six types of analyses for each construct (with listed options yes and no). Specifically, we asked whether participants analyzed (1) differences between independent groups (e.g., patients vs. non-patients) in the dynamics of the construct, (2) mean differences in the construct across independent groups (e.g., patients vs. non-patients), (3) differences between dependent groups (e.g., before vs. after intervention) in the dynamics of the construct, (4) mean differences in the construct across dependent groups (e.g., before vs. after intervention), (5) relationship(s) between the construct and other constructs (e.g., the correlation between depression and anxiety), and (6) a time trend in the construct. Participants were then asked to specify which type(s) of analyses they conducted with the construct to answer their research question.

Open Science Practices and Confidence

To assess open science practices among the participants in our sample, we asked them for each construct separately whether they reported all analysis steps in detail, such that the analyses could be reproduced if one had access to the data and if the syntax or code for all analysis steps is publicly available, for example, on OSF. Additionally, participants were asked to rate their confidence level that their analyses for the construct (including any assessment of reliability, factor structure, and measurement invariance) were ideal for their research design. Finally, participants were given the option to provide any additional comments related to the study in a final open text box.

Codebook

Variable name	Label	Values
duration	Duration (in seconds)	Numeric
finished	Finished	True<br>False
studies_designed	How many intensive longitudinal data (ILD) studies have you (co-)designed (approximately)?	Numeric (integer)

Variable name	Label	Values
studies_analyzed	How many intensive longitudinal data (ILD) studies have you (co-)analyzed (approximately)?	Numeric (integer)
knowledge_general	How would you rate your knowledge on the topic of measurement of psychological constructs in general?	Moderately knowledgeable<br>Very knowledgeable<br>Slightly knowledgeable<br>Not knowledgeable at all<br>Extremely knowledgeable
knowledge_ild	How would you rate your knowledge on the topic of measurement of psychological constructs in ILD studies?	Moderately knowledgeable<br>Very knowledgeable<br>Slightly knowledgeable<br>Not knowledgeable at all<br>Extremely knowledgeable
field	What field best describes your background?	Pedagogical sciences<br>Developmental psychology<br>Clinical psychology<br>Medical psychology<br>Cognitive psychology<br>Work and organizational psychology<br>Social psychology<br>Personality psychology<br>Organizational studies<br>Methodology and statistics<br>Epidemiology<br>Other, namely:
art_type	Please indicate which applies to your article.	Sent out for review and uploaded as a preprint<br>Published (with or without prior preprint<br>Sent out for review and not uploaded as a preprint
year_publish	In which year was the article accepted for publication?	Numeric (integer)

Variable name	Label	Values
year_review	In which year was the article submitted for review?	Numeric (integer)
year_preprint	In which year was the article uploaded as a preprint?	Numeric (integer)
position	What was your position at the time of analyzing the data?	Junior researcher<br>Ph.D. candidate<br>Postdoc<br>Senior researcher<br>Assistant professor<br>Associate professor<br>Full professor<br>Other namely:
collab	Did you collaborate with a methodologist, statistician, or psychometrician to analyze the data?	Yes<br>No<br>I don't know / I don't remember
n_subjects	What is the (approximate) number of subjects that you used for the analyses?	Numeric (integer)<br>I don't know / I don't remember
n_occasions	What is the (approximate) average number of measurement occasions per subject that you used for the analyses?	Numeric (integer)<br>I don't know / I don't remember
n_constructs	How many psychological constructs (e.g., depression) did you use in your study?<br>Note: only indicate constructs that were measured multiple times, and exclude constructs only measured once (e.g., at baseline).<br>Note: a psychological construct can be measured by one or multiple items.	Numeric (integer)<br>I don't know / I don't remember
c1_name	What was the first construct? (e.g., depression, anxiety, positive affect)	Character
c1_indep_dyn	Did you analyze differences between independent groups (e.g., patients vs. non-patients) in the dynamics of Construct 1?	Yes<br>No

Variable name	Label	Values
c1_indep_mean	Did you analyze mean differences in Construct 1 across independent groups (e.g., patients vs. non-patients)?	Yes<br>No
c1_dep_dyn	Did you analyze differences between dependent groups (e.g., before vs. after intervention) in the dynamics of Construct 1?	Yes<br>No
c1_dep_mean	Did you analyze mean differences in Construct 1 across dependent groups (e.g., before vs. after intervention)?	Yes<br>No
c1_other_constructs	Did you assess the relationship(s) between Construct 1 and other constructs (e.g., the correlation between depression and anxiety)?	Yes<br>No
c1_timetrend	Did you assess a time trend in Construct 1?	Yes<br>No
c1_analysis	Which type(s) of analyses did you conduct with Construct 1 to answer your research question?	Multilevel (V)AR model<br>ME-VAR (measurement error VAR)<br>Dynamic network models<br>Dynamic structural equation modeling (or dynamic factor analysis)<br>Latent growth curve modeling<br>Latent Markov modeling Latent difference scores<br>Differential equations<br>Multilevel regression<br>Multilevel SEM<br>Replicated time-series design<br>Mean square successive difference (MSSD)<br>State-space grid<br>Other, namely:
c1_n_items	What was the number of items that measured Construct 1?	Numeric (integer)<br>I don’t know / I don’t remember

Variable name	Label	Values
c1_construct_scores	How did you create construct scores for your analyses of Construct 1?	The construct scores were based on the scores on a single item<br>The construct scores were sum scores of multiple items<br>The construct scores were average scores across multiple items<br>The construct scores were factor scores obtained using a factor analysis model<br>The construct scores were factor scores obtained using an item response theory model<br>The construct scores were component scores obtained using principal component analysis<br>Other, namely:
c1_scale_prev	Did you use a scale to measure Construct 1 for which the reliability and/or factor structure have been evaluated in previous ILD studies?	Only reliability<br>Only factor structure<br>Reliability and factor structure<br>No; neither<br>I don't know / I don't remember
c1_scale_other	Did you use a scale to measure Construct 1 for which the reliability and/or factor structure have been evaluated in previous research other than ILD studies (e.g., cross-sectional studies)?	Only reliability<br>Only factor structure<br>Reliability and factor structure<br>No; neither<br>I don't know / I don't remember
c1_scale_mod	Did you modify the previously evaluated scale for Construct 1 in any way (e.g., removed, added, or translated items)?	Yes<br>No<br>I don't know / I don't remember
c1_scale_current	Did you evaluate the reliability and/or factor structure for the scale of Construct 1 in your current study?	Only reliability<br>Only factor structure<br>Reliability and factor structure<br>No; neither<br>I don't know / I don't remember

Variable name	Label	Values
c1_scale_rel	What were the reasons that you did not evaluate the reliability for the scale of Construct 1 in your current study?<br>Note: you can select multiple answers.	I did not know how<br>I did not know that it may be relevant for drawing valid inferences for my analyses<br>Other namely:
c1_scale_fac	What were the reasons that you did not evaluate the factor structure for the scale of Construct 1 in your current study<br>Note: you can select multiple answers	I did not know how<br>I did not know that it may be relevant for drawing valid inferences for my analyses<br>Other namely:
c1_item_prev	Did you use an item to measure Construct 1 for which the reliability has been evaluated in previous ILD studies?	Yes<br>No<br>I don't know / I don't remember
c1_item_other	Did you use an item to measure Construct 1 for which the reliability has been evaluated in previous research other than ILD studies (e.g., cross-sectional studies)?	Yes<br>No<br>I don't know / I don't remember
c1_item_mod	Did you modify the previously evaluated item for Construct 1 in any way (e.g., translated the item)?	Yes<br>No<br>I don't know / I don't remember
c1_item_cur	Did you use an item to measure Construct 1 for which the reliability has been evaluated in the current study?	Yes<br>No<br>I don't know / I don't remember
c1_item_rel	What were the reasons that you did not evaluate the reliability of the item of Construct 1 in your current study?<br>Note: you can select multiple answers	I did not know how<br>I did not know that it may be relevant for drawing valid inferences for my analyses<br>Other namely:
c1_me	Did you correct the construct scores of Construct 1 for measurement error?<br>Note: measurement error refers to all variation not due to actual variation in the psychological construct.	Yes<br>No<br>I don't know / I don't remember

Variable name	Label	Values
c1_mi	Did you assess some type(s) of measurement invariance for Construct 1 in your model?<br>Note: measurement invariance means that all systematic variability in item scores is attributable to the psychological construct of interest.	Yes<br>No<br>I don’t know / I don’t remember
c1_mi_no	What were the reasons that you did not assess measurement invariance for Construct 1?<br>Note: you can select multiple answers.	I did not know how<br>I did not know that it may be relevant for drawing valid inferences for my analyses<br>Other namely:
c1_mi_type	Which type(s) of measurement invariance did you assess for Construct 1?<br>Note: you can select multiple answers.	Invariance across subjects<br>Invariance across time<br>Invariance across subject- and/or time-specific covariates/groups<br>Invariance across the within-person and between-person levels<br>Other namely:
c1_mi_subjects	For invariance across subjects: What was the highest level of invariance that held for Construct 1?<br>Note: the levels are ordered, starting with the lowest level of invariance.	No level of invariance held<br>Configural invariance (i.e.whether the pattern of (non)zero factor loadings is invariant)<br>(Partial) loading invariance<br>(Partial) intercept/threshold invariance<br>(Partial) residual invariance<br>Other namely:
c1_mi_time	For invariance across time: What was the highest level of invariance that held for Construct 1?<br>Note: the levels are ordered, starting with the lowest level of invariance.	No level of invariance held<br>Configural invariance (i.e. whether the pattern of (non)zero factor loadings is invariant)<br>(Partial) loading invariance<br>(Partial) intercept/threshold invariance<br>(Partial) residual invariance<br>Other namely:

Variable name	Label	Values
c1_mi_covariate	For invariance across subject- and/or time-specific covariates/groups: What was the highest level of invariance that held for Construct 1?<br>Note: the levels are ordered, starting with the lowest level of invariance.	No level of invariance held<br>Configural invariance (i.e. whether the pattern of (non)zero factor loadings is invariant)<br>(Partial) loading invariance<br>(Partial) intercept/threshold invariance<br>(Partial) residual invariance<br>Other namely:
c1_mi_withinbetween	For invariance across the within-person and between-person levels: What was the highest level of invariance that held for Construct 1?<br>Note: the levels are ordered, starting with the lowest level of invariance.	No level of invariance held<br>Configural invariance (i.e. whether the pattern of (non)zero factor loadings is invariant)<br>(Partial) loading invariance<br>(Partial) intercept/threshold invariance<br>(Partial) residual invariance<br>Other namely:
c1_mi_other	You indicated you assessed an “other” type of invariance. What was the highest level of invariance that held for Construct 1?<br>Note: the levels are ordered, starting with the lowest level of invariance.	No level of invariance held<br>Configural invariance (i.e. whether the pattern of (non)zero factor loadings is invariant)<br>(Partial) loading invariance<br>(Partial) intercept/threshold invariance<br>(Partial) residual invariance<br>Other namely:
c1_mi_steps	You indicated for at least one type of measurement invariance that you did not have full measurement invariance.<br>Did you take follow-up steps to correct for not having full measurement invariance for Construct 1?	Yes<br>No
c1_mi_steps_yes	What were the follow-up steps you took to correct for not having full measurement invariance for Construct 1?	Character

Variable name	Label	Values
c1_mi_steps_no	What were the reasons that you did not take follow-up steps to correct for not having full measurement invariance for Construct 1?<br><br>Note: you can select multiple answers.	I did not know how<br><br>I did not know that it may be relevant for drawing valid inferences for my analyses<br><br>It was not necessary<br><br>Other namely:
c1_reproduce	Did you report all the analysis steps in such detail that the reader could reproduce the analyses if they had the data?<br><br>Note: the analysis steps include, if applicable, scale construction, assessment of reliability, factor structure, and measurement invariance for Construct 1.	Yes<br><br>No
c1_code	Is the syntax or code for all the analysis steps publicly available (e.g., on the Open Science Framework)?<br><br>Note: the analysis steps include, if applicable, scale construction, assessment of reliability, factor structure, and measurement invariance for Construct 1.	Yes<br><br>No
c1_confidence	How confident are you that you analyses for Construct 1 (including any assessment of reliability, factor structure and measurement invariance) were ideal for your research design?	Not at all confident<br><br>Slightly confident<br><br>Moderately confident<br><br>Very confident<br><br>Extremely confident
c2_name	What was the second construct? (e.g., depression, anxiety, positive affect)	Character
c2_indep_dyn	Did you analyze differences between independent groups (e.g., patients vs. non-patients) in the dynamics of Construct 2?	Yes<br><br>No
c2_indep_mean	Did you analyze mean differences in Construct 2 across independent groups (e.g., patients vs. non-patients)?	Yes<br><br>No
c2_dep_dyn	Did you analyze differences between dependent groups (e.g., before vs. after intervention) in the dynamics of Construct 2?	Yes<br><br>No

Variable name	Label	Values
c2_dep_mean	Did you analyze mean differences in Construct 2 across dependent groups (e.g., before vs. after intervention)?	Yes<br>No
c2_other_constructs	Did you assess the relationship(s) between Construct 2 and other constructs (e.g., the correlation between depression and anxiety)?	Yes<br>No
c2_timetrend	Did you assess a time trend in Construct 2?	Yes<br>No
c2_analysis	Which type(s) of analyses did you conduct with Construct 2 to answer your research question?	Multilevel (V)AR model<br>ME-VAR (measurement error VAR)<br>Dynamic network models<br>Dynamic structural equation modeling (or dynamic factor analysis)<br>Latent growth curve modeling<br>Latent Markov modeling Latent difference scores<br>Differential equations<br>Multilevel regression<br>Multilevel SEM<br>Replicated time-series design<br>Mean square successive difference (MSSD)<br>State-space grid<br>Other, namely:
c2_n_items	What was the number of items that measured Construct 2?	Numeric (integer)<br>I don’t know / I don’t remember

Variable name	Label	Values
c2_construct_scores	How did you create construct scores for your analyses of Construct 2?	The construct scores were based on the scores on a single item<br>The construct scores were sum scores of multiple items<br>The construct scores were average scores across multiple items<br>The construct scores were factor scores obtained using a factor analysis model<br>The construct scores were factor scores obtained using an item response theory model<br>The construct scores were component scores obtained using principal component analysis<br>Other, namely:
c2_scale_val_prev	Did you use a scale to measure Construct 2 for which the reliability and/or factor structure have been evaluated in previous ILD studies?	Only reliability<br>Only factor structure<br>Reliability and factor structure<br>No; neither<br>I don’t know / I don’t remember
c2_scale_other	Did you use a scale to measure Construct 2 for which the reliability and/or factor structure have been evaluated in previous research other than ILD studies (e.g., cross-sectional studies)?	Only reliability<br>Only factor structure<br>Reliability and factor structure<br>No; neither<br>I don’t know / I don’t remember
c2_scale_mod	Did you modify the previously evaluated scale for Construct 2 in any way (e.g., removed, added, or translated items)?	Yes<br>No<br>I don’t know / I don’t remember
c2_scale_val_cur	Did you evaluate the reliability and/or factor structure for the scale of Construct 2 in your current study?	Only reliability<br>Only factor structure<br>Reliability and factor structure<br>No; neither<br>I don’t know / I don’t remember

Variable name	Label	Values
c2_scale_rel	What were the reasons that you did not evaluate the reliability for the scale of Construct 2 in your current study?<br>Note: you can select multiple answers.	I did not know how<br>I did not know that it may be relevant for drawing valid inferences for my analyses<br>Other namely:
c2_scale_fac	What were the reasons that you did not evaluate the factor structure for the scale of Construct 2 in your current study<br>Note: you can select multiple answers	I did not know how<br>I did not know that it may be relevant for drawing valid inferences for my analyses<br>Other namely:
c2_item_val_prev	Did you use an item to measure Construct 2 for which the reliability has been evaluated in previous ILD studies?	Yes<br>No<br>I don't know / I don't remember
c2_item_val_other	Did you use an item to measure Construct 2 for which the reliability has been evaluated in previous research other than ILD studies (e.g., cross-sectional studies)?	Yes<br>No<br>I don't know / I don't remember
c2_item_mod	Did you modify the previously evaluated item for Construct 2 in any way (e.g., translated the item)?	Yes<br>No<br>I don't know / I don't remember
c2_item_val_cur	Did you use an item to measure Construct 2 for which the reliability has been evaluated in the current study?	Yes<br>No<br>I don't know / I don't remember
c2_item_rel	What were the reasons that you did not evaluate the reliability of the item of Construct 2 in your current study?<br>Note: you can select multiple answers	I did not know how<br>I did not know that it may be relevant for drawing valid inferences for my analyses<br>Other namely:
c2_me	Did you correct the construct scores of Construct 2 for measurement error?<br>Note: measurement error refers to all variation not due to actual variation in the psychological construct.	Yes<br>No<br>I don't know / I don't remember

Variable name	Label	Values
c2_mi	Did you assess some type(s) of measurement invariance for Construct 2 in your model?<br><br>Note: measurement invariance means that all systematic variability in item scores is attributable to the psychological construct of interest.	Yes<br><br>No<br><br>I don’t know / I don’t remember
c2_mi_no	What were the reasons that you did not assess measurement invariance for Construct 2?<br><br>Note: you can select multiple answers.	I did not know how<br><br>I did not know that it may be relevant for drawing valid inferences for my analyses<br><br>Other namely:
c2_mi_type	Which type(s) of measurement invariance did you assess for Construct 2?<br><br>Note: you can select multiple answers.	Invariance across subjects<br><br>Invariance across time<br><br>Invariance across subject- and/or time-specific covariates/groups<br><br>Invariance across the within-person and between-person levels<br><br>Other namely:
c2_mi_subjects	For invariance across subjects: What was the highest level of invariance that held for Construct 2?<br><br>Note: the levels are ordered, starting with the lowest level of invariance.	No level of invariance held<br><br>Configural invariance (i.e.whether the pattern of (non)zero factor loadings is invariant)<br><br>(Partial) loading invariance<br><br>(Partial) intercept/threshold invariance<br><br>(Partial) residual invariance<br><br>Other namely:
c2_mi_time	For invariance across time: What was the highest level of invariance that held for Construct 2?<br><br>Note: the levels are ordered, starting with the lowest level of invariance.	No level of invariance held<br><br>Configural invariance (i.e. whether the pattern of (non)zero factor loadings is invariant)<br><br>(Partial) loading invariance<br><br>(Partial) intercept/threshold invariance<br><br>(Partial) residual invariance<br><br>Other namely:

Variable name	Label	Values
c2_mi_covariate	For invariance across subject- and/or time-specific covariates/groups: What was the highest level of invariance that held for Construct 2?<br><br>Note: the levels are ordered, starting with the lowest level of invariance.	No level of invariance held<br><br>Configural invariance (i.e. whether the pattern of (non)zero factor loadings is invariant)<br><br>(Partial) loading invariance<br><br>(Partial) intercept/threshold invariance<br><br>(Partial) residual invariance<br><br>Other namely:
c2_mi_withinbetween	For invariance across the within-person and between-person levels: What was the highest level of invariance that held for Construct 2?<br><br>Note: the levels are ordered, starting with the lowest level of invariance.	No level of invariance held<br><br>Configural invariance (i.e. whether the pattern of (non)zero factor loadings is invariant)<br><br>(Partial) loading invariance<br><br>(Partial) intercept/threshold invariance<br><br>(Partial) residual invariance<br><br>Other namely:
c2_mi_other	You indicated you assessed an “other” type of invariance. What was the highest level of invariance that held for Construct 2?<br><br>Note: the levels are ordered, starting with the lowest level of invariance.	No level of invariance held<br><br>Configural invariance (i.e. whether the pattern of (non)zero factor loadings is invariant)<br><br>(Partial) loading invariance<br><br>(Partial) intercept/threshold invariance<br><br>(Partial) residual invariance<br><br>Other namely:
c2_mi_steps	You indicated for at least one type of measurement invariance that you did not have full measurement invariance.<br><br>Did you take follow-up steps to correct for not having full measurement invariance for Construct 2?	Yes<br><br>No
c2_mi_steps_yes	What were the follow-up steps you took to correct for not having full measurement invariance for Construct 2?	Character

Variable name	Label	Values
c2_mi_steps_no	What were the reasons that you did not take follow-up steps to correct for not having full measurement invariance for Construct 2?<br><br>Note: you can select multiple answers.	I did not know how<br><br>I did not know that it may be relevant for drawing valid inferences for my analyses<br><br>It was not necessary<br><br>Other namely:
c2_reproduce	Did you report all the analysis steps in such detail that the reader could reproduce the analyses if they had the data?<br><br>Note: the analysis steps include, if applicable, scale construction, assessment of reliability, factor structure, and measurement invariance for Construct 2.	Yes<br><br>No
c2_code	Is the syntax or code for all the analysis steps publicly available (e.g., on the Open Science Framework)?<br><br>Note: the analysis steps include, if applicable, scale construction, assessment of reliability, factor structure, and measurement invariance for Construct 2.	Yes<br><br>No
c2_confidence	How confident are you that you analyses for Construct 2 (including any assessment of reliability, factor structure and measurement invariance) were ideal for your research design?	Not at all confident<br><br>Slightly confident<br><br>Moderately confident<br><br>Very confident<br><br>Extremely confident
