# Supplementary material for: Assessing and accounting for measurement in intensive longitudinal studies: current practices, considerations, and avenues for improvement
Source: Qual Life Res. 2024 Jun 13;33(8):2107–18. doi: 10.1007/s11136-024-03678-0 (PMC11286633; doi:10.1007/s11136-024-03678-0)
Supplement: Supplementary file 3 — Supplementary file3 (HTML 1059 KB) [file 11136_2024_3678_MOESM3_ESM.html]

Supplemental Material C


# Supplemental Material C

### Results for ‘Assessing and Accounting for Measurement in Intensive Longitudinal Studies: Current Practices, Considerations, and Avenues for Improvement’

# **Survey info**

## Duration

The number of seconds it took the respondent to complete the survey.
This is the entire duration of the response; if a respondent stops in
the middle of the survey, closes the browser, and comes back another
day, that time is counted.

| participants | responses | missing | distinct | min | median | mean | max |
| --- | --- | --- | --- | --- | --- | --- | --- |
| 42 | 42 | 0 | 41 | 106 | 467.5 | 8978.67 | 351668 |

## Finished

The Finished column details whether the response was submitted or
closed. A “1” or “True” indicates the respondent reached an end point in
their survey. A “0” or “False” indicates the respondent left their
survey before reaching an end point and the response was instead closed
manually or due to session expiration.

| participants | responses | missing | distinct | min | median | mean | max |
| --- | --- | --- | --- | --- | --- | --- | --- |
| 42 | 42 | 0 | 2 | 0 | 1 | 0.67 | 1 |

|  |  |  |
| --- | --- | --- |
| Value | False | True |
| Freq | 14 | 28 |
| Perc | 33.3 | 66.7 |

# **Expertise and Background**

## How many intensive longitudinal data (ILD) studies have you (co-)designed (approximately)?

| participants | responses | missing | distinct | min | median | mean | max |
| --- | --- | --- | --- | --- | --- | --- | --- |
| 42 | 42 | 0 | 9 | 0 | 2 | 3.29 | 40 |

|  |  |  |  |  |  |  |  |  |  |
| --- | --- | --- | --- | --- | --- | --- | --- | --- | --- |
| Value | 0 | 1 | 2 | 3 | 4 | 5 | 6 | 7 | 40 |
| Freq | 6 | 7 | 13 | 6 | 3 | 3 | 1 | 2 | 1 |
| Perc | 14.3 | 16.7 | 31 | 14.3 | 7.1 | 7.1 | 2.4 | 4.8 | 2.4 |

## How many intensive longitudinal data (ILD) studies have you (co-)analyzed (approximately)?

| participants | responses | missing | distinct | min | median | mean | max |
| --- | --- | --- | --- | --- | --- | --- | --- |
| 42 | 42 | 0 | 12 | 1 | 4 | 5.4 | 40 |

|  |  |  |  |  |  |  |  |  |  |  |  |  |
| --- | --- | --- | --- | --- | --- | --- | --- | --- | --- | --- | --- | --- |
| Value | 1 | 2 | 3 | 4 | 5 | 6 | 7 | 8 | 9 | 12 | 15 | 40 |
| Freq | 6 | 9 | 4 | 5 | 7 | 2 | 1 | 1 | 3 | 1 | 2 | 1 |
| Perc | 14.3 | 21.4 | 9.5 | 11.9 | 16.7 | 4.8 | 2.4 | 2.4 | 7.1 | 2.4 | 4.8 | 2.4 |

## How would you rate your knowledge on the topic of measurement of psychological constructs in *general*?

- Not knowledgeable at all = 1
- Moderately knowledgeable = 3
- Extremely knowledgeable = 5

| participants | responses | missing | distinct | min | median | mean | max |
| --- | --- | --- | --- | --- | --- | --- | --- |
| 42 | 42 | 0 | 5 | 1 | 3 | 3.29 | 5 |

|  |  |  |  |  |  |
| --- | --- | --- | --- | --- | --- |
| Value | Not knowledgeable at all | Slightly knowledgeable | Moderately knowledgeable | Very knowledgeable | Extremely knowledgeable |
| Freq | 1 | 4 | 23 | 10 | 4 |
| Perc | 2.4 | 9.5 | 54.8 | 23.8 | 9.5 |

## How would you rate your knowledge on the topic of measurement of psychological constructs in *ILD studies*?

- Not knowledgeable at all = 1
- Moderately knowledgeable = 3
- Extremely knowledgeable = 5

| participants | responses | missing | distinct | min | median | mean | max |
| --- | --- | --- | --- | --- | --- | --- | --- |
| 42 | 42 | 0 | 4 | 2 | 3 | 3.19 | 5 |

|  |  |  |  |  |  |
| --- | --- | --- | --- | --- | --- |
| Value | Not knowledgeable at all | Slightly knowledgeable | Moderately knowledgeable | Very knowledgeable | Extremely knowledgeable |
| Freq | 0 | 7 | 22 | 11 | 2 |
| Perc | 0 | 16.7 | 52.4 | 26.2 | 4.8 |

## What field best describes your background?

Responses were provided by 42 participants (missing: 0). More than
one answer could be indicated. The 42 participants provided a total of
61 responses.

|  | Freq | Perc |
| --- | --- | --- |
| Methodology and statistics | 14 | 23.0 |
| Clinical psychology | 12 | 19.7 |
| Developmental psychology | 7 | 11.5 |
| Social psychology | 5 | 8.2 |
| Personality psychology | 5 | 8.2 |
| Work and organizational psychology | 3 | 4.9 |
| Pedagogical sciences | 2 | 3.3 |
| Medical psychology | 1 | 1.6 |
| Cognitive psychology | 1 | 1.6 |
| Organizational studies | 1 | 1.6 |
| Epidemiology | 1 | 1.6 |
| Other namely: Behavioral Economics | 1 | 1.6 |
| Other namely: Communication Science | 1 | 1.6 |
| Other namely: Economics | 1 | 1.6 |
| Other namely: Educational psychology | 1 | 1.6 |
| Other namely: Health psychology | 1 | 1.6 |
| Other namely: Health Psychology | 1 | 1.6 |
| Other namely: Kinesiology | 1 | 1.6 |
| Other namely: Media psychology | 1 | 1.6 |
| Other namely: Neuropsychology | 1 | 1.6 |

# **Article information**

## Please indicate which applies to your article.

| participants | responses | missing | distinct | min | median | mean | max |
| --- | --- | --- | --- | --- | --- | --- | --- |
| 42 | 41 | 1 | 3 | NA | NA | NA | NA |

|  |  |  |  |
| --- | --- | --- | --- |
| Value | Published (with or without prior preprint) | Sent out for review and not uploaded as a preprint | Sent out for review and uploaded as a preprint |
| Freq | 25 | 9 | 7 |
| Perc | 61 | 22 | 17.1 |

## In which year was the article accepted for publication?

| participants | responses | missing | distinct | min | median | mean | max |
| --- | --- | --- | --- | --- | --- | --- | --- |
| 42 | 21 | 21 | 6 | 2013 | 2021 | 2020.24 | 2022 |

|  |  |  |  |  |  |  |
| --- | --- | --- | --- | --- | --- | --- |
| Value | 2013 | 2018 | 2019 | 2020 | 2021 | 2022 |
| Freq | 1 | 1 | 3 | 5 | 5 | 6 |
| Perc | 4.8 | 4.8 | 14.3 | 23.8 | 23.8 | 28.6 |

## In which year was the article submitted for review?

| participants | responses | missing | distinct | min | median | mean | max |
| --- | --- | --- | --- | --- | --- | --- | --- |
| 42 | 16 | 26 | 2 | 2021 | 2022 | 2021.81 | 2022 |

|  |  |  |
| --- | --- | --- |
| Value | 2021 | 2022 |
| Freq | 3 | 13 |
| Perc | 18.8 | 81.2 |

## In which year was the article uploaded as a preprint?

No participants indicated *Uploaded as a preprint and not sent out
for review* in the *Please indicate which applies to your
article* question.

## What was your position at the time of analyzing the data?

| participants | responses | missing | distinct | min | median | mean | max |
| --- | --- | --- | --- | --- | --- | --- | --- |
| 42 | 37 | 5 | 9 | NA | NA | NA | NA |

|  |  |  |  |  |  |  |  |  |  |
| --- | --- | --- | --- | --- | --- | --- | --- | --- | --- |
| Value | Assistant professor | Associate professor | Full professor | Junior researcher | Other namely: | Other namely: Assistant Research Professor | Ph.D. candidate | Postdoc | Senior researcher |
| Freq | 4 | 3 | 2 | 2 | 1 | 1 | 14 | 6 | 4 |
| Perc | 10.8 | 8.1 | 5.4 | 5.4 | 2.7 | 2.7 | 37.8 | 16.2 | 10.8 |

## Did you collaborate with a methodologist, statistician, or psychometrician to analyze the data?

- No = 0
- Yes = 1

| participants | responses | missing | distinct | min | median | mean | max |
| --- | --- | --- | --- | --- | --- | --- | --- |
| 42 | 26 | 16 | 2 | 0 | 0 | 0.12 | 1 |

|  |  |  |
| --- | --- | --- |
| Value | No | Yes |
| Freq | 23 | 3 |
| Perc | 88.5 | 11.5 |

## Field / Collaboration crosstabs

Of the 28 participants with no background in Methodology and
Statistics, 3 indicated that they collaborated with a methodologist,
statistician, or psychometrician to analyze the data.

# **Analysis information**

## What is the (approximate) number of subjects that you used for the analyses?

| participants | responses | missing | distinct | min | median | mean | max |
| --- | --- | --- | --- | --- | --- | --- | --- |
| 42 | 35 | 7 | 30 | 1 | 90 | 665.97 | 10000 |

|  |  |  |  |  |  |  |  |  |  |  |  |  |  |  |  |  |  |  |  |  |  |  |  |  |  |  |  |  |  |  |
| --- | --- | --- | --- | --- | --- | --- | --- | --- | --- | --- | --- | --- | --- | --- | --- | --- | --- | --- | --- | --- | --- | --- | --- | --- | --- | --- | --- | --- | --- | --- |
| Value | 1 | 10 | 24 | 35 | 41 | 47 | 50 | 53 | 62 | 72 | 79 | 80 | 89 | 90 | 100 | 114 | 120 | 123 | 124 | 127 | 142 | 158 | 226 | 230 | 296 | 300 | 372 | 3000 | 7000 | 10000 |
| Freq | 4 | 1 | 1 | 1 | 2 | 1 | 1 | 1 | 1 | 1 | 1 | 1 | 1 | 1 | 2 | 1 | 1 | 1 | 1 | 1 | 1 | 1 | 1 | 1 | 1 | 1 | 1 | 1 | 1 | 1 |
| Perc | 11.4 | 2.9 | 2.9 | 2.9 | 5.7 | 2.9 | 2.9 | 2.9 | 2.9 | 2.9 | 2.9 | 2.9 | 2.9 | 2.9 | 5.7 | 2.9 | 2.9 | 2.9 | 2.9 | 2.9 | 2.9 | 2.9 | 2.9 | 2.9 | 2.9 | 2.9 | 2.9 | 2.9 | 2.9 | 2.9 |

## What is the (approximate) average number of measurement occasions per subject that you used for the analyses?

| participants | responses | missing | distinct | min | median | mean | max |
| --- | --- | --- | --- | --- | --- | --- | --- |
| 42 | 34 | 8 | 27 | 3 | 70 | 320.85 | 4037 |

|  |  |  |  |  |  |  |  |  |  |  |  |  |  |  |  |  |  |  |  |  |  |  |  |  |  |  |  |
| --- | --- | --- | --- | --- | --- | --- | --- | --- | --- | --- | --- | --- | --- | --- | --- | --- | --- | --- | --- | --- | --- | --- | --- | --- | --- | --- | --- |
| Value | 3 | 5 | 12 | 23 | 25 | 28 | 30 | 35 | 43 | 52 | 54 | 56 | 60 | 70 | 80 | 95 | 100 | 110 | 150 | 160 | 286 | 445 | 522 | 540 | 1000 | 1480 | 4037 |
| Freq | 1 | 1 | 2 | 1 | 1 | 1 | 1 | 1 | 1 | 1 | 1 | 2 | 2 | 2 | 1 | 1 | 2 | 2 | 1 | 1 | 1 | 1 | 1 | 1 | 2 | 1 | 1 |
| Perc | 2.9 | 2.9 | 5.9 | 2.9 | 2.9 | 2.9 | 2.9 | 2.9 | 2.9 | 2.9 | 2.9 | 5.9 | 5.9 | 5.9 | 2.9 | 2.9 | 5.9 | 5.9 | 2.9 | 2.9 | 2.9 | 2.9 | 2.9 | 2.9 | 5.9 | 2.9 | 2.9 |

## How many psychological constructs (e.g., depression) did you use in your study?

| participants | responses | missing | distinct | min | median | mean | max |
| --- | --- | --- | --- | --- | --- | --- | --- |
| 42 | 36 | 6 | 11 | 1 | 3 | 4.31 | 15 |

|  |  |  |  |  |  |  |  |  |  |  |  |
| --- | --- | --- | --- | --- | --- | --- | --- | --- | --- | --- | --- |
| Value | 1 | 2 | 3 | 5 | 6 | 7 | 8 | 9 | 10 | 13 | 15 |
| Freq | 7 | 8 | 5 | 6 | 3 | 2 | 1 | 1 | 1 | 1 | 1 |
| Perc | 19.4 | 22.2 | 13.9 | 16.7 | 8.3 | 5.6 | 2.8 | 2.8 | 2.8 | 2.8 | 2.8 |

## What was the construct? (e.g., depression, anxiety, positive affect)

Responses were provided by 37 participants (missing: 5). Participants
could indicate a maximum of two constructs. The 37 participants
indicated a total of 62 constructs.

|  | x |
| --- | --- |
| Affect | 17 |
| (Acceptance of) fatigue | 3 |
| Depression / Feeling down | 3 |
| (Perceived) stress | 2 |
| Arousal | 2 |
| Drinking willingness | 2 |
| Loneliness | 2 |
| Personality | 2 |
| self-esteem | 2 |
| Anxiety | 1 |
| body surveillance | 1 |
| Coping efficacy | 1 |
| Emotional exhaustion | 1 |
| emotions | 1 |
| Gender minority resilience (identity pride, community connectedness) | 1 |
| goal conflict | 1 |
| Grattitude | 1 |
| Guilt | 1 |
| Hostility | 1 |
| Intention | 1 |
| Irritability | 1 |
| Mood | 1 |
| Optimistic expectations | 1 |
| Parental criticism | 1 |
| Parental warmth | 1 |
| Perseverence | 1 |
| Pessimistic expectations | 1 |
| Planning | 1 |
| pride | 1 |
| processing speed | 1 |
| Purpose in life | 1 |
| Repetitive negative thinking | 1 |
| smoking habit | 1 |
| Social media use | 1 |
| Suicidal ideation | 1 |
| valence | 1 |

Please note that from this point forward, the unit of analysis is the
number of constructs (62 constructs).

## Did you analyze differences between independent groups (e.g., patients vs. non-patients) in the dynamics of the construct?

- No = 0
- Yes = 1

| constructs | responses | missing | distinct | min | median | mean | max |
| --- | --- | --- | --- | --- | --- | --- | --- |
| 62 | 56 | 6 | 2 | 0 | 0 | 0.16 | 1 |

|  |  |  |
| --- | --- | --- |
| Value | No | Yes |
| Freq | 47 | 9 |
| Perc | 83.9 | 16.1 |

## Did you analyze mean differences in the construct across independent groups (e.g., patients vs. non-patients)?

- No = 0
- Yes = 1

| constructs | responses | missing | distinct | min | median | mean | max |
| --- | --- | --- | --- | --- | --- | --- | --- |
| 62 | 56 | 6 | 2 | 0 | 0 | 0.12 | 1 |

|  |  |  |
| --- | --- | --- |
| Value | No | Yes |
| Freq | 49 | 7 |
| Perc | 87.5 | 12.5 |

## Did you analyze differences between dependent groups (e.g., before vs. after intervention) in the dynamics of the construct?

- No = 0
- Yes = 1

| constructs | responses | missing | distinct | min | median | mean | max |
| --- | --- | --- | --- | --- | --- | --- | --- |
| 62 | 56 | 6 | 2 | 0 | 0 | 0.05 | 1 |

|  |  |  |
| --- | --- | --- |
| Value | No | Yes |
| Freq | 53 | 3 |
| Perc | 94.6 | 5.4 |

## Did you analyze mean differences in the construct across dependent groups (e.g., before vs. after intervention)?

- No = 0
- Yes = 1

| constructs | responses | missing | distinct | min | median | mean | max |
| --- | --- | --- | --- | --- | --- | --- | --- |
| 62 | 56 | 6 | 2 | 0 | 0 | 0.05 | 1 |

|  |  |  |
| --- | --- | --- |
| Value | No | Yes |
| Freq | 53 | 3 |
| Perc | 94.6 | 5.4 |

## Did you assess the relationship(s) between the construct and other constructs (e.g., the correlation between depression and anxiety)?

- No = 0
- Yes = 1

| constructs | responses | missing | distinct | min | median | mean | max |
| --- | --- | --- | --- | --- | --- | --- | --- |
| 62 | 56 | 6 | 2 | 0 | 1 | 0.77 | 1 |

|  |  |  |
| --- | --- | --- |
| Value | No | Yes |
| Freq | 13 | 43 |
| Perc | 23.2 | 76.8 |

## Did you assess a time trend in the construct?

- No = 0
- Yes = 1

| constructs | responses | missing | distinct | min | median | mean | max |
| --- | --- | --- | --- | --- | --- | --- | --- |
| 62 | 56 | 6 | 2 | 0 | 1 | 0.57 | 1 |

|  |  |  |
| --- | --- | --- |
| Value | No | Yes |
| Freq | 24 | 32 |
| Perc | 42.9 | 57.1 |

## Which type(s) of analyses did you conduct with the construct to answer your research question?

### All constructs

Responses were provided for 56 out of 62 constructs (missing: 6
constructs).

More than one answer could be indicated per construct. For 56
constructs, a total of 84 responses were provided.

|  | Freq | Perc |
| --- | --- | --- |
| Multilevel regression | 25 | 29.8 |
| Multilevel (V)AR model | 12 | 14.3 |
| Dynamic structural equation modeling (or dynamic factor analysis) | 9 | 10.7 |
| Mean square successive difference (MSSD) | 6 | 7.1 |
| Latent growth curve modeling | 5 | 6.0 |
| Dynamic network models | 4 | 4.8 |
| Multilevel SEM | 4 | 4.8 |
| Replicated time-series design | 2 | 2.4 |
| Other namely: Exponentially Weighted Moving Average (EWMA) control charts | 2 | 2.4 |
| Other namely: Within-Between Model | 2 | 2.4 |
| Other namely: Calculated SD rMSSD autocorrelation and then used multiple regression to link positive affect those to depression | 1 | 1.2 |
| Other namely: Dynamic Item Response Theory | 1 | 1.2 |
| Other namely: generalized additive models | 1 | 1.2 |
| Other namely: Generalized addtive models | 1 | 1.2 |
| Other namely: Mixed-effect location scale model | 1 | 1.2 |
| Other namely: Multi-level mixed effect models | 1 | 1.2 |
| Other namely: None of the above (resampling approach) | 1 | 1.2 |
| Other namely: Repeated n=1 moving window estimates of the autocorrelation and variance | 1 | 1.2 |
| Other namely: Repeated single-subject analyses of trends in moving-window estimated autocorrelation and variance | 1 | 1.2 |
| Other namely: Same as positive affect | 1 | 1.2 |
| Other namely: this was a tutorial | 1 | 1.2 |
| Other namely: Time varying autoregressive | 1 | 1.2 |
| Other namely: used multiple regression to link negative affect to depression | 1 | 1.2 |

### Constructs measured by more than one item

Responses were provided for 26 out of 26 constructs that had more
than one item (missing: 0).

More than one answer could be indicated per construct. For 26
constructs that had more than one item, a total of 41 responses were
provided.

|  | Freq | Perc |
| --- | --- | --- |
| Multilevel regression | 12 | 29.3 |
| Multilevel (V)AR model | 5 | 12.2 |
| Mean square successive difference (MSSD) | 4 | 9.8 |
| Latent growth curve modeling | 3 | 7.3 |
| Dynamic structural equation modeling (or dynamic factor analysis) | 2 | 4.9 |
| Other namely: Exponentially Weighted Moving Average (EWMA) control charts | 2 | 4.9 |
| Multilevel SEM | 2 | 4.9 |
| Dynamic network models | 1 | 2.4 |
| Replicated time-series design | 1 | 2.4 |
| Other namely: Calculated SD rMSSD autocorrelation and then used multiple regression to link positive affect those to depression | 1 | 2.4 |
| Other namely: Dynamic Item Response Theory | 1 | 2.4 |
| Other namely: generalized additive models | 1 | 2.4 |
| Other namely: Multi-level mixed effect models | 1 | 2.4 |
| Other namely: Repeated n=1 moving window estimates of the autocorrelation and variance | 1 | 2.4 |
| Other namely: Same as positive affect | 1 | 2.4 |
| Other namely: this was a tutorial | 1 | 2.4 |
| Other namely: Time varying autoregressive | 1 | 2.4 |
| Other namely: used multiple regression to link negative affect to depression | 1 | 2.4 |

### Constructs measured by one item

Responses were provided for 29 out of 29 constructs measured by one
item (missing: 0).

More than one answer could be indicated per construct. For 29
constructs that were measured by one item, a total of 42 responses were
provided.

|  | Freq | Perc |
| --- | --- | --- |
| Multilevel regression | 13 | 31.0 |
| Dynamic structural equation modeling (or dynamic factor analysis) | 7 | 16.7 |
| Multilevel (V)AR model | 6 | 14.3 |
| Dynamic network models | 3 | 7.1 |
| Latent growth curve modeling | 2 | 4.8 |
| Mean square successive difference (MSSD) | 2 | 4.8 |
| Other namely: Within-Between Model | 2 | 4.8 |
| Multilevel SEM | 2 | 4.8 |
| Replicated time-series design | 1 | 2.4 |
| Other namely: Generalized addtive models | 1 | 2.4 |
| Other namely: Mixed-effect location scale model | 1 | 2.4 |
| Other namely: None of the above (resampling approach) | 1 | 2.4 |
| Other namely: Repeated single-subject analyses of trends in moving-window estimated autocorrelation and variance | 1 | 2.4 |

## What was the number of items that measured the construct?

| constructs | responses | missing | distinct | min | median | mean | max |
| --- | --- | --- | --- | --- | --- | --- | --- |
| 62 | 53 | 9 | 10 | 1 | 1 | 2.56 | 15 |

|  |  |  |  |  |  |  |  |  |  |  |
| --- | --- | --- | --- | --- | --- | --- | --- | --- | --- | --- |
| Value | 1 | 2 | 2.5 | 3 | 4 | 5 | 6 | 9 | 10 | 15 |
| Freq | 29 | 5 | 1 | 9 | 3 | 1 | 1 | 1 | 2 | 1 |
| Perc | 54.7 | 9.4 | 1.9 | 17 | 5.7 | 1.9 | 1.9 | 1.9 | 3.8 | 1.9 |

## How did you create construct scores for your analyses of the construct?

| constructs | responses | missing | distinct | min | median | mean | max |
| --- | --- | --- | --- | --- | --- | --- | --- |
| 62 | 55 | 7 | 9 | NA | NA | NA | NA |

|  |  |  |  |  |  |  |  |  |  |
| --- | --- | --- | --- | --- | --- | --- | --- | --- | --- |
| Value | Other namely: first pca then sum scores | Other namely: modeled latent variable directly with SEM | Other namely: Same PA | Other namely: We assessed all items separately | The construct scores were average scores across multiple items | The construct scores were based on the scores on a single item | The construct scores were factor scores obtained using a factor analysis model | The construct scores were factor scores obtained using an item response theory model | The construct scores were sum scores of multiple items |
| Freq | 1 | 1 | 1 | 1 | 16 | 29 | 2 | 2 | 2 |
| Perc | 1.8 | 1.8 | 1.8 | 1.8 | 29.1 | 52.7 | 3.6 | 3.6 | 3.6 |

# **Scale validation**

Note that the scale validation questions were presented if
respondents selected any of the following answers in response to the
*How did you create construct scores* question:

- The construct scores were sum scores of multiple items
- The construct scores were average scores across multiple items
- The construct scores were factor scores obtained using a factor
  analysis model
- The construct scores were factor scores obtained using an item
  response theory model
- The construct scores were component scores obtained using principal
  component analysis

Total: 26 constructs measured by scales.

## Did you use a scale to measure the construct for which the reliability and/or factor structure have been evaluated in previous ILD studies?

| scales | responses | missing | distinct | min | median | mean | max |
| --- | --- | --- | --- | --- | --- | --- | --- |
| 26 | 20 | 6 | 3 | NA | NA | NA | NA |

|  |  |  |  |
| --- | --- | --- | --- |
| Value | No, neither | Yes, only reliability | Yes, reliability and factor structure |
| Freq | 7 | 2 | 11 |
| Perc | 35 | 10 | 55 |

## Did you use a scale to measure the construct for which the reliability and/or factor structure have been evaluated in previous research other than ILD studies (e.g., cross-sectional studies)?

| scales | responses | missing | distinct | min | median | mean | max |
| --- | --- | --- | --- | --- | --- | --- | --- |
| 26 | 14 | 12 | 2 | NA | NA | NA | NA |

|  |  |  |
| --- | --- | --- |
| Value | No, neither | Yes, reliability and factor structure |
| Freq | 7 | 7 |
| Perc | 50 | 50 |

## Did you modify the previously evaluated scale for the construct in any way (e.g., removed, added, or translated items)?

- No = 0
- Yes = 1

| scales | responses | missing | distinct | min | median | mean | max |
| --- | --- | --- | --- | --- | --- | --- | --- |
| 26 | 13 | 13 | 2 | 0 | 0 | 0.46 | 1 |

|  |  |  |
| --- | --- | --- |
| Value | No | Yes |
| Freq | 7 | 6 |
| Perc | 53.8 | 46.2 |

## Did you evaluate the reliability and/or factor structure for the scale of the construct in your current study?

Number of scales that indicated *Yes, reliability and factor
structure* in more than one context (for example, in both a previous
ILD study and the current study): 7.

| scales | responses | missing | distinct | min | median | mean | max |
| --- | --- | --- | --- | --- | --- | --- | --- |
| 26 | 22 | 4 | 3 | NA | NA | NA | NA |

|  |  |  |  |
| --- | --- | --- | --- |
| Value | No, neither | Yes, only reliability | Yes, reliability and factor structure |
| Freq | 4 | 10 | 8 |
| Perc | 18.2 | 45.5 | 36.4 |

## What were the reasons that you did not evaluate the reliability for the scale of the construct in your current study?

Note that this question was presented if respondents selected
*Yes, only factor structure* or *No, neither* in response
to the *Did you evaluate the reliability and/or factor structure for
the scale of the construct in your current study?* question.

Responses were provided for 4 out of 4 scales (missing: 0 scales).
More than one answer could be indicated per scale. For 4 scales, a total
of 4 responses were provided.

| scales | responses | missing | distinct | min | median | mean | max |
| --- | --- | --- | --- | --- | --- | --- | --- |
| 4 | 4 | 0 | 2 | NA | NA | NA | NA |

|  |  |  |
| --- | --- | --- |
| Value | I did not know how | I did not know that it may be relevant for drawing valid inferences for my analyses |
| Freq | 2 | 2 |
| Perc | 50 | 50 |

## What were the reasons that you did not evaluate the factor structure for the scale of the construct in your current study?

Note that this question was presented if respondents selected
*Yes, only reliability* or *No, neither* in response to
the *Did you evaluate the reliability and/or factor structure for the
scale of the construct in your current study?* question.

Responses were provided for 14 out of 14 scales (missing: 0 scales).
More than one answer could be indicated per scale. For 14 scales, a
total of 16 responses were provided.

|  | Freq | Perc |
| --- | --- | --- |
| I did not know that it may be relevant for drawing valid inferences for my analyses | 5 | 31.2 |
| I did not know how | 4 | 25.0 |
| Other namely: 2 items is not sufficient for factor structure | 1 | 6.2 |
| Other namely: Chose not to. | 1 | 6.2 |
| Other namely: Did not think it was necessary for the tutorial; | 1 | 6.2 |
| Other namely: I know how but chose not to. | 1 | 6.2 |
| Other namely: I thought that Cronbach’s alpha would determine reliability by showing how the items load on the same factor? | 1 | 6.2 |
| Other namely: I was using a set of items from the web as an example. | 1 | 6.2 |
| Other namely: Three items were not deemed enough to examine the factor structure | 1 | 6.2 |

## Scale modification crosstabs

If the scale was modified (rows), was the scale subsequently
evaluated in the current study (columns)?

|  | No, neither | Yes, only reliability | Yes, reliability and factor structure | Total |
| --- | --- | --- | --- | --- |
| Modified | 0 | 4 | 2 | 6 |
| Not modified | 2 | 2 | 3 | 7 |
| Total | 2 | 6 | 5 | 13 |

## Scale evaluation crosstabs

Number of scales that were evaluated in a previous ILD study,
previous other (non-ILD) study, or in the current study. Note that rows
that end up with a count of 0 have been removed from the table.

| Previous ILD study | Previous other study | Current study | Count |
| --- | --- | --- | --- |
| No, neither | Yes, reliability and factor structure | No, neither | 2 |
| Yes, only reliability | Yes, reliability and factor structure | No, neither | 1 |
| Yes, reliability and factor structure | NA | No, neither | 1 |
| No, neither | No, neither | Yes, only reliability | 3 |
| Yes, reliability and factor structure | No, neither | Yes, only reliability | 1 |
| No, neither | Yes, reliability and factor structure | Yes, only reliability | 1 |
| Yes, only reliability | Yes, reliability and factor structure | Yes, only reliability | 1 |
| Yes, reliability and factor structure | Yes, reliability and factor structure | Yes, only reliability | 2 |
| Yes, reliability and factor structure | NA | Yes, only reliability | 2 |
| No, neither | No, neither | Yes, reliability and factor structure | 1 |
| Yes, reliability and factor structure | No, neither | Yes, reliability and factor structure | 2 |
| Yes, reliability and factor structure | NA | Yes, reliability and factor structure | 3 |
| NA | NA | Yes, reliability and factor structure | 2 |
| NA | NA | NA | 4 |

# **Item validation**

Note that the item validation questions were presented if respondents
selected *The construct scores were based on the scores on a single
item* in response to the *How did you create construct
scores* question.

Total: 29 constructs measured by one item.

## Did you use an item to measure the construct for which the reliability has been evaluated in previous ILD studies?

- No = 0
- Yes = 1

| items | responses | missing | distinct | min | median | mean | max |
| --- | --- | --- | --- | --- | --- | --- | --- |
| 29 | 26 | 3 | 2 | 0 | 0 | 0.31 | 1 |

|  |  |  |
| --- | --- | --- |
| Value | No | Yes |
| Freq | 18 | 8 |
| Perc | 69.2 | 30.8 |

## Did you use an item to measure the construct for which the reliability has been evaluated in previous research other than ILD studies (e.g., cross-sectional studies)?

- No = 0
- Yes = 1

| items | responses | missing | distinct | min | median | mean | max |
| --- | --- | --- | --- | --- | --- | --- | --- |
| 29 | 25 | 4 | 2 | 0 | 1 | 0.6 | 1 |

|  |  |  |
| --- | --- | --- |
| Value | No | Yes |
| Freq | 10 | 15 |
| Perc | 40 | 60 |

## Did you modify the previously evaluated item for the construct in any way (e.g., translated the item)?

- No = 0
- Yes = 1

| items | responses | missing | distinct | min | median | mean | max |
| --- | --- | --- | --- | --- | --- | --- | --- |
| 29 | 6 | 23 | 2 | 0 | 0 | 0.33 | 1 |

|  |  |  |
| --- | --- | --- |
| Value | No | Yes |
| Freq | 4 | 2 |
| Perc | 66.7 | 33.3 |

## Did you use an item to measure the construct for which the reliability has been evaluated in the current study?

- No = 0
- Yes = 1

| items | responses | missing | distinct | min | median | mean | max |
| --- | --- | --- | --- | --- | --- | --- | --- |
| 29 | 28 | 1 | 2 | 0 | 0 | 0.21 | 1 |

|  |  |  |
| --- | --- | --- |
| Value | No | Yes |
| Freq | 22 | 6 |
| Perc | 78.6 | 21.4 |

## What were the reasons that you did not evaluate the reliability of the item of the construct in your current study?

Note that this question was presented if respondents selected
*No* in response to the *Did you evaluate the reliability of
the item in your current study?* question.

Responses were provided for 22 out of 22 items (missing: 0 items).
More than one answer could be indicated per item. For 22 items, a total
of 24 responses were provided.

|  | Freq | Perc |
| --- | --- | --- |
| I did not know how | 10 | 41.7 |
| I did not know that it may be relevant for drawing valid inferences for my analyses | 4 | 16.7 |
| Other namely: 1- we only had 1 item. 2- other non-ild studies evaluated it. 3- the measure was very explicit and direct. | 1 | 4.2 |
| Other namely: Any time I do it reviewers ask me to remove the information from the manuscript for space | 1 | 4.2 |
| Other namely: Common methods for single-item reliability in ESM studies did not seem appropriate given our research question since we explicitly do not consider Loneliness as a trait. | 1 | 4.2 |
| Other namely: Illustrative analysis in which no conclusion about the effect of the constructs under investigation were drawb | 1 | 4.2 |
| Other namely: Illustrative example analysis not used to draw conclusions regarding effect of depression | 1 | 4.2 |
| Other namely: it was a single item | 1 | 4.2 |
| Other namely: It was not feasible with the kind of data avialable | 1 | 4.2 |
| Other namely: Main focus was to reduce patient burden and patient understanding of the item. | 1 | 4.2 |
| Other namely: Only 1 item already evaluated with non-ild studies direct explicit question | 1 | 4.2 |
| Other namely: Whenever I include such information in my manuscript reviewers ask it to be removed and my supplement for this manuscript was already 30 pages | 1 | 4.2 |

## Item modification crosstabs

If the item was modified (rows), was the item subsequently evaluated
for reliability in the current study (columns)?

|  | No | Yes | Total |
| --- | --- | --- | --- |
| Modified | 2 | 0 | 2 |
| Not modified | 2 | 2 | 4 |
| Total | 4 | 2 | 6 |

## Item evaluation crosstabs

Number of items that were evaluated in a previous ILD study, previous
other (non-ILD) study, or in the current study. Note that rows that end
up with a count of 0 have been removed from the table.

- Number of items that assessed reliability in more than one context
  (for example, in both a previous ILD study and the current study):
  8.
- Number of items that did not assess reliability in any context
  (i.e., previous ILD, previous non-ILD, and current study): 8.
- Number of items that were not validated in a previous study but were
  validated in the current study: 2.

| Previous ILD study | Previous other study | Current study | Count |
| --- | --- | --- | --- |
| N | N | N | 8 |
| N | Y | N | 5 |
| Y | Y | N | 4 |
| NA | Y | N | 2 |
| N | NA | N | 1 |
| Y | NA | N | 2 |
| N | N | Y | 2 |
| N | Y | Y | 2 |
| Y | Y | Y | 2 |
| NA | NA | NA | 1 |

# **Measurement error and invariance**

## Did you correct the construct scores of the construct for measurement error?

- No = 0
- Yes = 1

Note that this question was presented if respondents selected any of
the following answers in response to the *How did you create
construct score* question.

- The construct scores were based on the scores on a single item
- The construct scores were sum scores of multiple items
- The construct scores were average scores across multiple items
- Other, namely..

Number of constructs: 51.

| constructs | responses | missing | distinct | min | median | mean | max |
| --- | --- | --- | --- | --- | --- | --- | --- |
| 51 | 46 | 5 | 2 | 0 | 0 | 0.07 | 1 |

|  |  |  |
| --- | --- | --- |
| Value | No | Yes |
| Freq | 43 | 3 |
| Perc | 93.5 | 6.5 |

## Did you assess some type(s) of measurement invariance for the construct in your model?

- No = 0
- Yes = 1

Note that this question was presented if respondents selected any of
the following answers in response to the *How did you create
construct score* question.

- The construct scores were sum scores of multiple items
- The construct scores were average scores across multiple items
- The construct scores were factor scores obtained using a factor
  analysis model
- The construct scores were factor scores obtained using an item
  response theory model
- The construct scores were component scores obtained using principal
  component analysis
- Other, namely..

Number of constructs: 26

| constructs | responses | missing | distinct | min | median | mean | max |
| --- | --- | --- | --- | --- | --- | --- | --- |
| 26 | 25 | 1 | 2 | 0 | 0 | 0.16 | 1 |

|  |  |  |
| --- | --- | --- |
| Value | No | Yes |
| Freq | 21 | 4 |
| Perc | 84 | 16 |

## What were the reasons that you did not assess measurement invariance for the construct?

Note that this question was presented if respondents selected
*No* in response to the *Did you assess some type(s) of
measurement invariance for the construct in your model?*
question.

Responses were provided for 21 out of 21 constructs (missing: 0
constructs). More than one answer could be indicated per construct. For
21 constructs, a total of 27 responses were provided.

|  | Freq | Perc |
| --- | --- | --- |
| I did not know how | 7 | 25.9 |
| I did not know that it may be relevant for drawing valid inferences for my analyses | 6 | 22.2 |
| Other namely: Again we examined change over time for each subject separately as we were not examining fluctuations measurement invariance is not an issue | 1 | 3.7 |
| Other namely: Analyses were already quite a lot and did not know how | 1 | 3.7 |
| Other namely: Analyses were already quite a lot and did not know the best way to do it. | 1 | 3.7 |
| Other namely: As the purpose of the analysis was to propose a new technique we did not considered assessing measurement invariance neccessary and also tools to assess measurement invarianc | 1 | 3.7 |
| Other namely: Chose not to | 1 | 3.7 |
| Other namely: Chose not to. | 1 | 3.7 |
| Other namely: No time | 1 | 3.7 |
| Other namely: not applicable | 1 | 3.7 |
| Other namely: Not necessary for purposes of demonstration | 1 | 3.7 |
| Other namely: Not the focus of my question | 1 | 3.7 |
| Other namely: See previous answer | 1 | 3.7 |
| Other namely: The model was already very complex | 1 | 3.7 |
| Other namely: This was a tutorial and this was not relevant | 1 | 3.7 |
| Other namely: We were examining change over time for each individual separately so measurement invariance is not a problem in these type of analyses | 1 | 3.7 |

## Which type(s) of measurement invariance did you assess for the construct?

Note that this question was presented if respondents selected
*Yes* in response to the *Which type(s) of measurement
invariance did you assess for the construct?* question.

Responses were provided for 4 out of 4 constructs (missing: 0
constructs). More than one answer could be indicated per construct. For
4 constructs, a total of 10 responses were provided.

|  | Freq | Perc |
| --- | --- | --- |
| Invariance across subjects | 4 | 40 |
| Invariance across time | 2 | 20 |
| Invariance across subject- and/or time-specific covariates/groups | 2 | 20 |
| Invariance across the within-person and between-person levels | 2 | 20 |

## For invariance across subjects: What was the highest level of invariance that held for the construct?

Note that this question was presented if respondents selected
*invariance across subjects* in response to the *Which type(s)
of measurement invariance did you assess for the construct?*
question.

| constructs | responses | missing | distinct | min | median | mean | max |
| --- | --- | --- | --- | --- | --- | --- | --- |
| 4 | 4 | 0 | 3 | NA | NA | NA | NA |

|  |  |  |  |
| --- | --- | --- | --- |
| Value | (Partial) intercept/threshold invariance | (Partial) loading invariance | Configural invariance (i.e. whether the pattern of (non)zero factor loadings is invariant) |
| Freq | 1 | 2 | 1 |
| Perc | 25 | 50 | 25 |

## For invariance across time: What was the highest level of invariance that held for the construct?

Note that this question was presented if respondents selected
*invariance across time* in response to the *Which type(s) of
measurement invariance did you assess for the construct?*
question.

| constructs | responses | missing | distinct | min | median | mean | max |
| --- | --- | --- | --- | --- | --- | --- | --- |
| 4 | 2 | 2 | 1 | NA | NA | NA | NA |

|  |  |
| --- | --- |
| Value | (Partial) intercept/threshold invariance |
| Freq | 2 |
| Perc | 100 |

## For invariance across subject- and/or time-specific covariates/groups: What was the highest level of invariance that held for the construct?

Note that this question was presented if respondents selected
*invariance across subject- and/or time-specific
covariates/groups* in response to the *Which type(s) of
measurement invariance did you assess for the construct?*
question.

| constructs | responses | missing | distinct | min | median | mean | max |
| --- | --- | --- | --- | --- | --- | --- | --- |
| 4 | 2 | 2 | 1 | NA | NA | NA | NA |

|  |  |
| --- | --- |
| Value | (Partial) loading invariance |
| Freq | 2 |
| Perc | 100 |

## For invariance across the within-person and between-person levels: What was the highest level of invariance that held for the construct?

Note that this question was presented if respondents selected
*invariance across the within-person and between-person levels*
in response to the *Which type(s) of measurement invariance did you
assess for the construct?* question.

| constructs | responses | missing | distinct | min | median | mean | max |
| --- | --- | --- | --- | --- | --- | --- | --- |
| 4 | 2 | 2 | 1 | NA | NA | NA | NA |

|  |  |
| --- | --- |
| Value | (Partial) loading invariance |
| Freq | 2 |
| Perc | 100 |

## You indicated you assessed an “other” type of invariance. What was the highest level of invariance that held for the construct?

No participants indicated *Other type of invariance* in the
*Which type(s) of measurement invariance did you assess*
question.

## You indicated for at least one type of measurement invariance that you did not have full measurement invariance. Did you take follow-up steps to correct for not having full measurement invariance for the construct?

- No = 0
- Yes = 1

| constructs | responses | missing | distinct | min | median | mean | max |
| --- | --- | --- | --- | --- | --- | --- | --- |
| 4 | 4 | 0 | 2 | 0 | 0.5 | 0.5 | 1 |

|  |  |  |
| --- | --- | --- |
| Value | No | Yes |
| Freq | 2 | 2 |
| Perc | 50 | 50 |

## What were the follow-up steps you took to correct for not having full measurement invariance for the construct?

Note that this question was presented if respondents selected
*Yes* in response to the *Did you take follow-up steps to
correct for not having full measurement invariance for the
construct?* question.

| constructs | responses | missing | distinct | min | median | mean | max |
| --- | --- | --- | --- | --- | --- | --- | --- |
| 2 | 1 | 1 | 1 | NA | NA | NA | NA |

|  |  |
| --- | --- |
| Value | random effects on item parameters |
| Freq | 1 |
| Perc | 100 |

## What were the reasons that you did not take follow-up steps to correct for not having full measurement invariance for the construct?

Note that this question was presented if respondents selected
*No* in response to the *Did you take follow-up steps to
correct for not having full measurement invariance for the
construct?* question.

| constructs | responses | missing | distinct | min | median | mean | max |
| --- | --- | --- | --- | --- | --- | --- | --- |
| 2 | 2 | 0 | 1 | NA | NA | NA | NA |

|  |  |
| --- | --- |
| Value | I did not know that it may be relevant for drawing valid inferences for my analyses |
| Freq | 2 |
| Perc | 100 |

# **MI assessed crosstabs**

Rows indicate type of analysis. Columns indicate if measurement
invariance was tested.


|  | I don’t know / I don’t remember | No | Yes | NA | Total |
| --- | --- | --- | --- | --- | --- |
| Dynamics independent groups | 0 | 2 | 4 | 3 | 9 |
| Means independent groups | 0 | 1 | 3 | 3 | 7 |
| Dynamics dependent groups | 0 | 0 | 1 | 2 | 3 |
| Means dependent groups | 0 | 0 | 1 | 2 | 3 |
| Relationship with other constructs | 1 | 12 | 4 | 26 | 43 |
| Time trend | 1 | 13 | 4 | 14 | 32 |
| Total | 2 | 28 | 17 | 50 | 97 |

# **Open Science Practices**

Note that the following questions were presented once for every
construct.

## Did you report all the analysis steps in such detail that the reader could reproduce the analyses if they had the data?

- No = 0
- Yes = 1

| constructs | responses | missing | distinct | min | median | mean | max |
| --- | --- | --- | --- | --- | --- | --- | --- |
| 62 | 54 | 8 | 2 | 0 | 1 | 0.93 | 1 |

|  |  |  |
| --- | --- | --- |
| Value | No | Yes |
| Freq | 4 | 50 |
| Perc | 7.4 | 92.6 |

## Is the syntax or code for all the analysis steps publicly available (e.g., on the Open Science Framework)?

- No = 0
- Yes = 1

| constructs | responses | missing | distinct | min | median | mean | max |
| --- | --- | --- | --- | --- | --- | --- | --- |
| 62 | 54 | 8 | 2 | 0 | 1 | 0.61 | 1 |

|  |  |  |
| --- | --- | --- |
| Value | No | Yes |
| Freq | 21 | 33 |
| Perc | 38.9 | 61.1 |

## How confident are you that you analyses for the construct (including any assessment of reliability, factor structure and measurement invariance) were ideal for your research design?

- Not at all confident = 1
- Moderately confident = 3
- Extremely confident = 5

| constructs | responses | missing | distinct | min | median | mean | max |
| --- | --- | --- | --- | --- | --- | --- | --- |
| 62 | 54 | 8 | 5 | 1 | 3 | 2.94 | 5 |

|  |  |  |  |  |  |
| --- | --- | --- | --- | --- | --- |
| Value | Extremely confident | Moderately confident | Not at all confident | Slightly confident | Very confident |
| Freq | 5 | 25 | 5 | 11 | 8 |
| Perc | 9.3 | 46.3 | 9.3 | 20.4 | 14.8 |
