# Supplementary material for: Assessing and accounting for measurement in intensive longitudinal studies: current practices, considerations, and avenues for improvement
Source: Qual Life Res. 2024 Jun 13;33(8):2107–18. doi: 10.1007/s11136-024-03678-0 (PMC11286633; doi:10.1007/s11136-024-03678-0)
Supplement: Supplementary file 4 — Supplementary file4 (PDF 235 KB) [file 11136_2024_3678_MOESM4_ESM.pdf]

Supplemental Material C

Results for 'Assessing and Accounting for Measurement in Intensive Longitudinal Studies: Current Practices, Considerations, and Avenues for Improvement'

Contents

Survey info 3

Duration . . . 3

Finished . . . 4

Expertise and Background 4

How many intensive longitudinal data (ILD) studies have you (co-)designed (approximately)? . . . 4

How many intensive longitudinal data (ILD) studies have you (co-)analyzed (approximately)? . . . 4

How would you rate your knowledge on the topic of measurement of psychological constructs in *general*? . . . 4

How would you rate your knowledge on the topic of measurement of psychological constructs in *ILD studies*? . . . 5

What field best describes your background? . . . 5

Article information 6

Please indicate which applies to your article. . . . 6

In which year was the article accepted for publication? . . . 6

In which year was the article submitted for review? . . . 6

In which year was the article uploaded as a preprint? . . . 6

What was your position at the time of analyzing the data? . . . 7

Did you collaborate with a methodologist, statistician, or psychometrician to analyze the data? . . . 7

Field / Collaboration crosstabs . . . 7

Analysis information 7

What is the (approximate) number of subjects that you used for the analyses? . . . 7

What is the (approximate) average number of measurement occasions per subject that you used for the analyses? . . . 8

How many psychological constructs (e.g., depression) did you use in your study? . . . 8

What was the construct? (e.g., depression, anxiety, positive affect) . . . 8

Did you analyze differences between independent groups (e.g., patients vs. non-patients) in the dynamics of the construct? . . . 9

Did you analyze mean differences in the construct across independent groups (e.g., patients vs. non-patients)? . . . 9

Did you analyze differences between dependent groups (e.g., before vs. after intervention) in the dynamics of the construct? . . . 10

Did you analyze mean differences in the construct across dependent groups (e.g., before vs. after intervention)? . . . 10

Did you assess the relationship(s) between the construct and other constructs (e.g., the correlation between depression and anxiety)? . . . 10

Did you assess a time trend in the construct? . . . 11

Which type(s) of analyses did you conduct with the construct to answer your research question? . 11

What was the number of items that measured the construct? . . . 13

How did you create construct scores for your analyses of the construct? . . . 13

Scale validation **13**

Did you use a scale to measure the construct for which the reliability and/or factor structure have been evaluated in previous ILD studies? . . . 13

Did you use a scale to measure the construct for which the reliability and/or factor structure have been evaluated in previous research other than ILD studies (e.g., cross-sectional studies)? . . 14

Did you modify the previously evaluated scale for the construct in any way (e.g., removed, added, or translated items)? . . . 14

Did you evaluate the reliability and/or factor structure for the scale of the construct in your current study? . . . 14

What were the reasons that you did not evaluate the reliability for the scale of the construct in your current study? . . . 15

What were the reasons that you did not evaluate the factor structure for the scale of the construct in your current study? . . . 15

Scale modification crosstabs . . . 15

Scale evaluation crosstabs . . . 16

Item validation **16**

Did you use an item to measure the construct for which the reliability has been evaluated in previous ILD studies? . . . 16

Did you use an item to measure the construct for which the reliability has been evaluated in previous research other than ILD studies (e.g., cross-sectional studies)? . . . 17

Did you modify the previously evaluated item for the construct in any way (e.g., translated the item)? . . . 17

Did you use an item to measure the construct for which the reliability has been evaluated in the current study? . . . 17

What were the reasons that you did not evaluate the reliability of the item of the construct in your current study? . . . 18

Item modification crosstabs . . . 18

Item evaluation crosstabs . . . 19

Measurement error and invariance 19

Did you correct the construct scores of the construct for measurement error? 19

Did you assess some type(s) of measurement invariance for the construct in your model? 20

What were the reasons that you did not assess measurement invariance for the construct? 20

Which type(s) of measurement invariance did you assess for the construct? 21

For invariance across subjects: What was the highest level of invariance that held for the construct? 21

For invariance across time: What was the highest level of invariance that held for the construct? . 21

For invariance across subject- and/or time-specific covariates/groups: What was the highest level of invariance that held for the construct? 22

For invariance across the within-person and between-person levels: What was the highest level of invariance that held for the construct? 22

You indicated you assessed an "other" type of invariance. What was the highest level of invariance that held for the construct? 22

You indicated for at least one type of measurement invariance that you did not have full measurement invariance. Did you take follow-up steps to correct for not having full measurement invariance for the construct? 22

What were the follow-up steps you took to correct for not having full measurement invariance for the construct? 23

What were the reasons that you did not take follow-up steps to correct for not having full measurement invariance for the construct? 23

MI assessed crosstabs 23

Open Science Practices 24

Did you report all the analysis steps in such detail that the reader could reproduce the analyses if they had the data? 24

Is the syntax or code for all the analysis steps publicly available (e.g., on the Open Science Framework)? 24

How confident are you that you analyses for the construct (including any assessment of reliability, factor structure and measurement invariance) were ideal for your research design? 24

Survey info

Duration

The number of seconds it took the respondent to complete the survey. This is the entire duration of the response; if a respondent stops in the middle of the survey, closes the browser, and comes back another day, that time is counted.

participants	responses	missing	distinct	min	median	mean	max
42	42	0	41	106	467.5	8978.67	351668

Finished

The Finished column details whether the response was submitted or closed. A "1" or "True" indicates the respondent reached an end point in their survey. A "0" or "False" indicates the respondent left their survey before reaching an end point and the response was instead closed manually or due to session expiration.

participants	responses	missing	distinct	min	median	mean	max
42	42	0	2	0	1	0.67	1

Value	False	True
Freq	14	28
Perc	33.3	66.7

Expertise and Background

How many intensive longitudinal data (ILD) studies have you (co-)designed (approximately)?

participants	responses	missing	distinct	min	median	mean	max
42	42	0	9	0	2	3.29	40

Value	0	1	2	3	4	5	6	7	40
Freq	6	7	13	6	3	3	1	2	1
Perc	14.3	16.7	31	14.3	7.1	7.1	2.4	4.8	2.4

How many intensive longitudinal data (ILD) studies have you (co-)analyzed (approximately)?

participants	responses	missing	distinct	min	median	mean	max
42	42	0	12	1	4	5.4	40

Value	1	2	3	4	5	6	7	8	9	12	15	40
Freq	6	9	4	5	7	2	1	1	3	1	2	1
Perc	14.3	21.4	9.5	11.9	16.7	4.8	2.4	2.4	7.1	2.4	4.8	2.4

How would you rate your knowledge on the topic of measurement of psychological constructs in *general*?

- Not knowledgeable at all = 1
- Moderately knowledgeable = 3
- Extremely knowledgeable = 5

participants	responses	missing	distinct	min	median	mean	max
42	42	0	5	1	3	3.29	5

Value	Not knowledgeable at all	Slightly knowledgeable	Moderately knowledgeable	Very knowledgeable	Extremely knowledgeable
Freq	1	4	23	10	4
Perc	2.4	9.5	54.8	23.8	9.5

How would you rate your knowledge on the topic of measurement of psychological constructs in *ILD studies*?

- Not knowledgeable at all = 1
- Moderately knowledgeable = 3
- Extremely knowledgeable = 5

participants	responses	missing	distinct	min	median	mean	max
42	42	0	4	2	3	3.19	5

Value	Not knowledgeable at all	Slightly knowledgeable	Moderately knowledgeable	Very knowledgeable	Extremely knowledgeable
Freq	0	7	22	11	2
Perc	0	16.7	52.4	26.2	4.8

What field best describes your background?

Responses were provided by 42 participants (missing: 0). More than one answer could be indicated. The 42 participants provided a total of 61 responses.

	Freq	Perc
Methodology and statistics	14	23.0
Clinical psychology	12	19.7
Developmental psychology	7	11.5
Social psychology	5	8.2
Personality psychology	5	8.2
Work and organizational psychology	3	4.9
Pedagogical sciences	2	3.3
Medical psychology	1	1.6
Cognitive psychology	1	1.6
Organizational studies	1	1.6
Epidemiology	1	1.6
Other namely: Behavioral Economics	1	1.6
Other namely: Communication Science	1	1.6
Other namely: Economics	1	1.6
Other namely: Educational psychology	1	1.6
Other namely: Health psychology	1	1.6

	Freq	Perc
Other namely: Health Psychology	1	1.6
Other namely: Kinesiology	1	1.6
Other namely: Media psychology	1	1.6
Other namely: Neuropsychology	1	1.6

Article information

Please indicate which applies to your article.

participants	responses	missing	distinct	min	median	mean	max
42	41	1	3	NA	NA	NA	NA

Value	Published (with or without prior preprint)	Sent out for review and not uploaded as a preprint	Sent out for review and uploaded as a preprint
Freq	25	9	7
Perc	61	22	17.1

In which year was the article accepted for publication?

participants	responses	missing	distinct	min	median	mean	max
42	21	21	6	2013	2021	2020.24	2022

Value	2013	2018	2019	2020	2021	2022
Freq	1	1	3	5	5	6
Perc	4.8	4.8	14.3	23.8	23.8	28.6

In which year was the article submitted for review?

participants	responses	missing	distinct	min	median	mean	max
42	16	26	2	2021	2022	2021.81	2022

Value	2021	2022
Freq	3	13
Perc	18.8	81.2

In which year was the article uploaded as a preprint?

No participants indicated *Uploaded as a preprint and not sent out for review* in the *Please indicate which applies to your article* question.

What was your position at the time of analyzing the data?

participants	responses	missing	distinct	min	median	mean	max
42	37	5	9	NA	NA	NA	NA

Value	Assistant professor	Associate professor	Full professor	Junior researcher	Other namely:	Other namely: Assistant Research Professor	Ph.D. candidate	Postdoc	Senior researcher
Freq	4	3	2	2	1	1	14	6	4
Perc	10.8	8.1	5.4	5.4	2.7	2.7	37.8	16.2	10.8

Did you collaborate with a methodologist, statistician, or psychometrician to analyze the data?

- No = 0
- Yes = 1

participants	responses	missing	distinct	min	median	mean	max
42	26	16	2	0	0	0.12	1

Value	No	Yes
Freq	23	3
Perc	88.5	11.5

Field / Collaboration crosstabs

Of the 28 participants with no background in Methodology and Statistics, 3 indicated that they collaborated with a methodologist, statistician, or psychometrician to analyze the data.

Analysis information

What is the (approximate) number of subjects that you used for the analyses?

participants	responses	missing	distinct	min	median	mean	max
42	35	7	30	1	90	665.97	10000

Value	1	10	24	35	41	47	50	53	62	72	79	80	89	90	100	114	120	123	124	127	142	158	226	230	296	300	372	3000	7000	10000
Freq	4	1	1	1	2	1	1	1	1	1	1	1	1	1	2	1	1	1	1	1	1	1	1	1	1	1	1	1	1	1
Perc	11.4	2.9	2.9	2.9	5.7	2.9	2.9	2.9	2.9	2.9	2.9	2.9	2.9	2.9	5.7	2.9	2.9	2.9	2.9	2.9	2.9	2.9	2.9	2.9	2.9	2.9	2.9	2.9	2.9	2.9

What is the (approximate) average number of measurement occasions per subject that you used for the analyses?

participants	responses	missing	distinct	min	median	mean	max
42	34	8	27	3	70	320.85	4037

Value	3	5	12	23	25	28	30	35	43	52	54	56	60	70	80	95	100	110	150	160	286	445	522	540	1000	1480	4037
Freq	1	1	2	1	1	1	1	1	1	1	1	2	2	2	1	1	2	2	1	1	1	1	1	1	2	1	1
Perc	2.9	2.9	5.9	2.9	2.9	2.9	2.9	2.9	2.9	2.9	2.9	5.9	5.9	5.9	2.9	2.9	5.9	5.9	2.9	2.9	2.9	2.9	2.9	2.9	5.9	2.9	2.9

How many psychological constructs (e.g., depression) did you use in your study?

participants	responses	missing	distinct	min	median	mean	max
42	36	6	11	1	3	4.31	15

Value	1	2	3	5	6	7	8	9	10	13	15
Freq	7	8	5	6	3	2	1	1	1	1	1
Perc	19.4	22.2	13.9	16.7	8.3	5.6	2.8	2.8	2.8	2.8	2.8

What was the construct? (e.g., depression, anxiety, positive affect)

Responses were provided by 37 participants (missing: 5). Participants could indicate a maximum of two constructs. The 37 participants indicated a total of 62 constructs.

	x
Affect	17
(Acceptance of) fatigue	3
Depression / Feeling down	3
(Perceived) stress	2
Arousal	2
Drinking willingness	2
Loneliness	2
Personality	2
self-esteem	2
Anxiety	1
body surveillance	1
Coping efficacy	1
Emotional exhaustion	1
emotions	1
Gender minority resilience (identity pride, community connectedness)	1
goal conflict	1
Grattitude	1
Guilt	1
Hostility	1
Intention	1

	x
Irritability	1
Mood	1
Optimistic expectations	1
Parental criticism	1
Parental warmth	1
Perseverence	1
Pessimistic expectations	1
Planning	1
pride	1
processing speed	1
Purpose in life	1
Repetitive negative thinking	1
smoking habit	1
Social media use	1
Suicidal ideation	1
valence	1

Please note that from this point forward, the unit of analysis is the number of constructs (62 constructs).

Did you analyze differences between independent groups (e.g., patients vs. non-patients) in the dynamics of the construct?

- No = 0
- Yes = 1

constructs	responses	missing	distinct	min	median	mean	max
62	56	6	2	0	0	0.16	1

Value	No	Yes
Freq	47	9
Perc	83.9	16.1

Did you analyze mean differences in the construct across independent groups (e.g., patients vs. non-patients)?

- No = 0
- Yes = 1

constructs	responses	missing	distinct	min	median	mean	max
62	56	6	2	0	0	0.12	1

Value	No	Yes
Freq	49	7

Perc	87.5	12.5

Did you analyze differences between dependent groups (e.g., before vs. after intervention) in the dynamics of the construct?

- No = 0
- Yes = 1

constructs	responses	missing	distinct	min	median	mean	max
62	56	6	2	0	0	0.05	1

Value	No	Yes
Freq	53	3
Perc	94.6	5.4

Did you analyze mean differences in the construct across dependent groups (e.g., before vs. after intervention)?

- No = 0
- Yes = 1

constructs	responses	missing	distinct	min	median	mean	max
62	56	6	2	0	0	0.05	1

Value	No	Yes
Freq	53	3
Perc	94.6	5.4

Did you assess the relationship(s) between the construct and other constructs (e.g., the correlation between depression and anxiety)?

- No = 0
- Yes = 1

constructs	responses	missing	distinct	min	median	mean	max
62	56	6	2	0	1	0.77	1

Value	No	Yes
Freq	13	43
Perc	23.2	76.8

Did you assess a time trend in the construct?

- No = 0
- Yes = 1

constructs	responses	missing	distinct	min	median	mean	max
62	56	6	2	0	1	0.57	1

Value	No	Yes
Freq	24	32
Perc	42.9	57.1

Which type(s) of analyses did you conduct with the construct to answer your research question?

All constructs

Responses were provided for 56 out of 62 constructs (missing: 6 constructs).

More than one answer could be indicated per construct. For 56 constructs, a total of 84 responses were provided.

	Freq	Perc
Multilevel regression	25	29.8
Multilevel (V)AR model	12	14.3
Dynamic structural equation modeling (or dynamic factor analysis)	9	10.7
Mean square successive difference (MSSD)	6	7.1
Latent growth curve modeling	5	6.0
Dynamic network models	4	4.8
Multilevel SEM	4	4.8
Replicated time-series design	2	2.4
Other namely: Exponentially Weighted Moving Average (EWMA) control charts	2	2.4
Other namely: Within-Between Model	2	2.4
Other namely: Calculated SD rMSSD autocorrelation and then used multiple regression to link positive affect those to depression	1	1.2
Other namely: Dynamic Item Response Theory	1	1.2
Other namely: generalized additive models	1	1.2
Other namely: Generalized addtive models	1	1.2
Other namely: Mixed-effect location scale model	1	1.2
Other namely: Multi-level mixed effect models	1	1.2
Other namely: None of the above (resampling approach)	1	1.2
Other namely: Repeated n=1 moving window estimates of the autocorrelation and variance	1	1.2
Other namely: Repeated single-subject analyses of trends in moving-window estimated autocorrelation and variance	1	1.2
Other namely: Same as positive affect	1	1.2
Other namely: this was a tutorial	1	1.2
Other namely: Time varying autoregressive	1	1.2
Other namely: used multiple regression to link negative affect to depression	1	1.2

Constructs measured by more than one item

Responses were provided for 26 out of 26 constructs that had more than one item (missing: 0).

More than one answer could be indicated per construct. For 26 constructs that had more than one item, a total of 41 responses were provided.

	Freq	Perc
Multilevel regression	12	29.3
Multilevel (V)AR model	5	12.2
Mean square successive difference (MSSD)	4	9.8
Latent growth curve modeling	3	7.3
Dynamic structural equation modeling (or dynamic factor analysis)	2	4.9
Other namely: Exponentially Weighted Moving Average (EWMA) control charts	2	4.9
Multilevel SEM	2	4.9
Dynamic network models	1	2.4
Replicated time-series design	1	2.4
Other namely: Calculated SD rMSSD autocorrelation and then used multiple regression to link positive affect those to depression	1	2.4
Other namely: Dynamic Item Response Theory	1	2.4
Other namely: generalized additive models	1	2.4
Other namely: Multi-level mixed effect models	1	2.4
Other namely: Repeated n=1 moving window estimates of the autocorrelation and variance	1	2.4
Other namely: Same as positive affect	1	2.4
Other namely: this was a tutorial	1	2.4
Other namely: Time varying autoregressive	1	2.4
Other namely: used multiple regression to link negative affect to depression	1	2.4

Constructs measured by one item

Responses were provided for 29 out of 29 constructs measured by one item (missing: 0).

More than one answer could be indicated per construct. For 29 constructs that were measured by one item, a total of 42 responses were provided.

	Freq	Perc
Multilevel regression	13	31.0
Dynamic structural equation modeling (or dynamic factor analysis)	7	16.7
Multilevel (V)AR model	6	14.3
Dynamic network models	3	7.1
Latent growth curve modeling	2	4.8
Mean square successive difference (MSSD)	2	4.8
Other namely: Within-Between Model	2	4.8
Multilevel SEM	2	4.8
Replicated time-series design	1	2.4
Other namely: Generalized addtive models	1	2.4
Other namely: Mixed-effect location scale model	1	2.4
Other namely: None of the above (resampling approach)	1	2.4
Other namely: Repeated single-subject analyses of trends in moving-window estimated autocorrelation and variance	1	2.4

What was the number of items that measured the construct?

constructs	responses	missing	distinct	min	median	mean	max
62	53	9	10	1	1	2.56	15

Value	1	2	2.5	3	4	5	6	9	10	15
Freq	29	5	1	9	3	1	1	1	2	1
Perc	54.7	9.4	1.9	17	5.7	1.9	1.9	1.9	3.8	1.9

How did you create construct scores for your analyses of the construct?

constructs	responses	missing	distinct	min	median	mean	max
62	55	7	9	NA	NA	NA	NA

Value	Other namely: first pca then sum scores	Other namely: modeled latent variable directly with SEM	Other namely: Same PA	Other namely: We assessed all items separately	The construct scores were average scores across multiple items	The construct scores were based on the scores on a single item	The construct scores were factor scores obtained using a factor analysis model	The construct scores were factor scores obtained using an item response theory model	The construct scores were sum scores of multiple items
Freq	1	1	1	1	16	29	2	2	2
Perc	1.8	1.8	1.8	1.8	29.1	52.7	3.6	3.6	3.6

Scale validation

Note that the scale validation questions were presented if respondents selected any of the following answers in response to the *How did you create construct scores* question:

- The construct scores were sum scores of multiple items
- The construct scores were average scores across multiple items
- The construct scores were factor scores obtained using a factor analysis model
- The construct scores were factor scores obtained using an item response theory model
- The construct scores were component scores obtained using principal component analysis

Total: 26 constructs measured by scales.

Did you use a scale to measure the construct for which the reliability and/or factor structure have been evaluated in previous ILD studies?

scales	responses	missing	distinct	min	median	mean	max
26	20	6	3	NA	NA	NA	NA

Value	No, neither	Yes, only reliability	Yes, reliability and factor structure
Freq	7	2	11
Perc	35	10	55

Did you use a scale to measure the construct for which the reliability and/or factor structure have been evaluated in previous research other than ILD studies (e.g., cross-sectional studies)?

scales	responses	missing	distinct	min	median	mean	max
26	14	12	2	NA	NA	NA	NA

Value	No, neither	Yes, reliability and factor structure
Freq	7	7
Perc	50	50

Did you modify the previously evaluated scale for the construct in any way (e.g., removed, added, or translated items)?

- No = 0
- Yes = 1

scales	responses	missing	distinct	min	median	mean	max
26	13	13	2	0	0	0.46	1

Value	No	Yes
Freq	7	6
Perc	53.8	46.2

Did you evaluate the reliability and/or factor structure for the scale of the construct in your current study?

Number of scales that indicated *Yes, reliability and factor structure* in more than one context (for example, in both a previous ILD study and the current study): 7.

scales	responses	missing	distinct	min	median	mean	max
26	22	4	3	NA	NA	NA	NA

Value	No, neither	Yes, only reliability	Yes, reliability and factor structure
Freq	4	10	8
Perc	18.2	45.5	36.4

What were the reasons that you did not evaluate the reliability for the scale of the construct in your current study?

Note that this question was presented if respondents selected *Yes, only factor structure* or *No, neither* in response to the *Did you evaluate the reliability and/or factor structure for the scale of the construct in your current study?* question.

Responses were provided for 4 out of 4 scales (missing: 0 scales). More than one answer could be indicated per scale. For 4 scales, a total of 4 responses were provided.

scales	responses	missing	distinct	min	median	mean	max
4	4	0	2	NA	NA	NA	NA

Value	I did not know how	I did not know that it may be relevant for drawing valid inferences for my analyses
Freq	2	2
Perc	50	50

What were the reasons that you did not evaluate the factor structure for the scale of the construct in your current study?

Note that this question was presented if respondents selected *Yes, only reliability* or *No, neither* in response to the *Did you evaluate the reliability and/or factor structure for the scale of the construct in your current study?* question.

Responses were provided for 14 out of 14 scales (missing: 0 scales). More than one answer could be indicated per scale. For 14 scales, a total of 16 responses were provided.

	Freq	Perc
I did not know that it may be relevant for drawing valid inferences for my analyses	5	31.2
I did not know how	4	25.0
Other namely: 2 items is not sufficient for factor structure	1	6.2
Other namely: Chose not to.	1	6.2
Other namely: Did not think it was necessary for the tutorial;	1	6.2
Other namely: I know how but chose not to.	1	6.2
Other namely: I thought that Cronbach's alpha would determine reliability by showing how the items load on the same factor?	1	6.2
Other namely: I was using a set of items from the web as an example.	1	6.2
Other namely: Three items were not deemed enough to examine the factor structure	1	6.2

Scale modification crosstabs

If the scale was modified (rows), was the scale subsequently evaluated in the current study (columns)?

	No, neither	Yes, only reliability	Yes, reliability and factor structure	Total
Modified	0	4	2	6
Not modified	2	2	3	7
Total	2	6	5	13

Scale evaluation crosstabs

Number of scales that were evaluated in a previous ILD study, previous other (non-ILD) study, or in the current study. Note that rows that end up with a count of 0 have been removed from the table.

Previous ILD study	Previous other study	Current study	Count
No, neither	Yes, reliability and factor structure	No, neither	2
Yes, only reliability	Yes, reliability and factor structure	No, neither	1
Yes, reliability and factor structure	NA	No, neither	1
No, neither	No, neither	Yes, only reliability	3
Yes, reliability and factor structure	No, neither	Yes, only reliability	1
No, neither	Yes, reliability and factor structure	Yes, only reliability	1
Yes, only reliability	Yes, reliability and factor structure	Yes, only reliability	1
Yes, reliability and factor structure	Yes, reliability and factor structure	Yes, only reliability	2
Yes, reliability and factor structure	NA	Yes, only reliability	2
No, neither	No, neither	Yes, reliability and factor structure	1
Yes, reliability and factor structure	No, neither	Yes, reliability and factor structure	2
Yes, reliability and factor structure	NA	Yes, reliability and factor structure	3
NA	NA	Yes, reliability and factor structure	2
NA	NA	NA	4

Item validation

Note that the item validation questions were presented if respondents selected *The construct scores were based on the scores on a single item* in response to the *How did you create construct scores* question.

Total: 29 constructs measured by one item.

Did you use an item to measure the construct for which the reliability has been evaluated in previous ILD studies?

- No = 0

- Yes = 1

items	responses	missing	distinct	min	median	mean	max
29	26	3	2	0	0	0.31	1

Value	No	Yes
Freq	18	8
Perc	69.2	30.8

Did you use an item to measure the construct for which the reliability has been evaluated in previous research other than ILD studies (e.g., cross-sectional studies)?

- No = 0
- Yes = 1

items	responses	missing	distinct	min	median	mean	max
29	25	4	2	0	1	0.6	1

Value	No	Yes
Freq	10	15
Perc	40	60

Did you modify the previously evaluated item for the construct in any way (e.g., translated the item)?

- No = 0
- Yes = 1

items	responses	missing	distinct	min	median	mean	max
29	6	23	2	0	0	0.33	1

Value	No	Yes
Freq	4	2
Perc	66.7	33.3

Did you use an item to measure the construct for which the reliability has been evaluated in the current study?

- No = 0
- Yes = 1

items	responses	missing	distinct	min	median	mean	max
29	28	1	2	0	0	0.21	1

Value	No	Yes
Freq	22	6
Perc	78.6	21.4

What were the reasons that you did not evaluate the reliability of the item of the construct in your current study?

Note that this question was presented if respondents selected *No* in response to the *Did you evaluate the reliability of the item in your current study?* question.

Responses were provided for 22 out of 22 items (missing: 0 items). More than one answer could be indicated per item. For 22 items, a total of 24 responses were provided.

	Freq	Perc
I did not know how	10	41.7
I did not know that it may be relevant for drawing valid inferences for my analyses	4	16.7
Other namely: 1- we only had 1 item. 2- other non-ild studies evaluated it. 3- the measure was very explicit and direct.	1	4.2
Other namely: Any time I do it reviewers ask me to remove the information from the manuscript for space	1	4.2
Other namely: Common methods for single-item reliability in ESM studies did not seem appropriate given our research question since we explicitly do not consider Loneliness as a trait.	1	4.2
Other namely: Illustrative analysis in which no conclusion about the effect of the constructs under investigation were drawb	1	4.2
Other namely: Illustrative example analysis not used to draw conclusions regarding effect of depression	1	4.2
Other namely: it was a single item	1	4.2
Other namely: It was not feasible with the kind of data avialable	1	4.2
Other namely: Main focus was to reduce patient burden and patient understanding of the item.	1	4.2
Other namely: Only 1 item already evaluated with non-ild studies direct explicit question	1	4.2
Other namely: Whenever I include such information in my manuscript reviewers ask it to be removed and my supplement for this manuscript was already 30 pages	1	4.2

Item modification crosstabs

If the item was modified (rows), was the item subsequently evaluated for reliability in the current study (columns)?

	No	Yes	Total
Modified	2	0	2
Not modified	2	2	4
Total	4	2	6

Item evaluation crosstabs

Number of items that were evaluated in a previous ILD study, previous other (non-ILD) study, or in the current study. Note that rows that end up with a count of 0 have been removed from the table.

- Number of items that assessed reliability in more than one context (for example, in both a previous ILD study and the current study): 8.
- Number of items that did not assess reliability in any context (i.e., previous ILD, previous non-ILD, and current study): 8.
- Number of items that were not validated in a previous study but were validated in the current study: 2.

Previous ILD study	Previous other study	Current study	Count
N	N	N	8
N	Y	N	5
Y	Y	N	4
NA	Y	N	2
N	NA	N	1
Y	NA	N	2
N	N	Y	2
N	Y	Y	2
Y	Y	Y	2
NA	NA	NA	1

Measurement error and invariance

Did you correct the construct scores of the construct for measurement error?

- No = 0
- Yes = 1

Note that this question was presented if respondents selected any of the following answers in response to the *How did you create construct score* question.

- The construct scores were based on the scores on a single item
- The construct scores were sum scores of multiple items
- The construct scores were average scores across multiple items
- Other, namely..

Number of constructs: 51.

constructs	responses	missing	distinct	min	median	mean	max
51	46	5	2	0	0	0.07	1

Value	No	Yes
Freq	43	3
Perc	93.5	6.5

Did you assess some type(s) of measurement invariance for the construct in your model?

- No = 0
- Yes = 1

Note that this question was presented if respondents selected any of the following answers in response to the *How did you create construct score* question.

- The construct scores were sum scores of multiple items
- The construct scores were average scores across multiple items
- The construct scores were factor scores obtained using a factor analysis model
- The construct scores were factor scores obtained using an item response theory model
- The construct scores were component scores obtained using principal component analysis
- Other, namely..

Number of constructs: 26

constructs	responses	missing	distinct	min	median	mean	max
26	25	1	2	0	0	0.16	1

Value	No	Yes
Freq	21	4
Perc	84	16

What were the reasons that you did not assess measurement invariance for the construct?

Note that this question was presented if respondents selected *No* in response to the *Did you assess some type(s) of measurement invariance for the construct in your model?* question.

Responses were provided for 21 out of 21 constructs (missing: 0 constructs). More than one answer could be indicated per construct. For 21 constructs, a total of 27 responses were provided.

	Freq	Perc
I did not know how	7	25.9
I did not know that it may be relevant for drawing valid inferences for my analyses	6	22.2
Other namely: Again we examined change over time for each subject separately as we were not examining fluctuations measurement invariance is not an issue	1	3.7
Other namely: Analyses were already quite a lot and did not know how	1	3.7
Other namely: Analyses were already quite a lot and did not know the best way to do it.	1	3.7
Other namely: As the purpose of the analysis was to propose a new technique we did not considered assessing measurement invariance neccessary and also tools to assess measurement invarianc	1	3.7
Other namely: Chose not to	1	3.7
Other namely: Chose not to.	1	3.7
Other namely: No time	1	3.7
Other namely: not applicable	1	3.7
Other namely: Not necessary for purposes of demonstration	1	3.7

	Freq	Perc
Other namely: Not the focus of my question	1	3.7
Other namely: See previous answer	1	3.7
Other namely: The model was already very complex	1	3.7
Other namely: This was a tutorial and this was not relevant	1	3.7
Other namely: We were examining change over time for each individual separately so measurement invariance is not a problem in these type of analyses	1	3.7

Which type(s) of measurement invariance did you assess for the construct?

Note that this question was presented if respondents selected *Yes* in response to the *Which type(s) of measurement invariance did you assess for the construct?* question.

Responses were provided for 4 out of 4 constructs (missing: 0 constructs). More than one answer could be indicated per construct. For 4 constructs, a total of 10 responses were provided.

	Freq	Perc
Invariance across subjects	4	40
Invariance across time	2	20
Invariance across subject- and/or time-specific covariates/groups	2	20
Invariance across the within-person and between-person levels	2	20

For invariance across subjects: What was the highest level of invariance that held for the construct?

Note that this question was presented if respondents selected *invariance across subjects* in response to the *Which type(s) of measurement invariance did you assess for the construct?* question.

constructs	responses	missing	distinct	min	median	mean	max
4	4	0	3	NA	NA	NA	NA

Value	(Partial) intercept/threshold invariance	(Partial) loading invariance	Configural invariance (i.e. whether the pattern of (non)zero factor loadings is invariant)
Freq	1	2	1
Perc	25	50	25

For invariance across time: What was the highest level of invariance that held for the construct?

Note that this question was presented if respondents selected *invariance across time* in response to the *Which type(s) of measurement invariance did you assess for the construct?* question.

constructs	responses	missing	distinct	min	median	mean	max
4	2	2	1	NA	NA	NA	NA

Value	(Partial) intercept/threshold invariance
Freq	2
Perc	100

For invariance across subject- and/or time-specific covariates/groups: What was the highest level of invariance that held for the construct?

Note that this question was presented if respondents selected *invariance across subject- and/or time-specific covariates/groups* in response to the *Which type(s) of measurement invariance did you assess for the construct?* question.

constructs	responses	missing	distinct	min	median	mean	max
4	2	2	1	NA	NA	NA	NA

Value	(Partial) loading invariance
Freq	2
Perc	100

For invariance across the within-person and between-person levels: What was the highest level of invariance that held for the construct?

Note that this question was presented if respondents selected *invariance across the within-person and between-person levels* in response to the *Which type(s) of measurement invariance did you assess for the construct?* question.

constructs	responses	missing	distinct	min	median	mean	max
4	2	2	1	NA	NA	NA	NA

Value	(Partial) loading invariance
Freq	2
Perc	100

You indicated you assessed an "other" type of invariance. What was the highest level of invariance that held for the construct?

No participants indicated *Other type of invariance* in the *Which type(s) of measurement invariance did you assess* question.

You indicated for at least one type of measurement invariance that you did not have full measurement invariance. Did you take follow-up steps to correct for not having full measurement invariance for the construct?

- No = 0
- Yes = 1

constructs	responses	missing	distinct	min	median	mean	max
4	4	0	2	0	0.5	0.5	1

Value	No	Yes
Freq	2	2
Perc	50	50

What were the follow-up steps you took to correct for not having full measurement invariance for the construct?

Note that this question was presented if respondents selected *Yes* in response to the *Did you take follow-up steps to correct for not having full measurement invariance for the construct?* question.

constructs	responses	missing	distinct	min	median	mean	max
2	1	1	1	NA	NA	NA	NA

Value	random effects on item parameters
Freq	1
Perc	100

What were the reasons that you did not take follow-up steps to correct for not having full measurement invariance for the construct?

Note that this question was presented if respondents selected *No* in response to the *Did you take follow-up steps to correct for not having full measurement invariance for the construct?* question.

constructs	responses	missing	distinct	min	median	mean	max
2	2	0	1	NA	NA	NA	NA

Value	I did not know that it may be relevant for drawing valid inferences for my analyses
Freq	2
Perc	100

MI assessed crosstabs

Table 93: Rows indicate type of analysis. Columns indicate if measurement invariance was tested.

	I don't know / I don't remember	No	Yes	NA	Total
Dynamics independent groups	0	2	4	3	9
Means independent groups	0	1	3	3	7
Dynamics dependent groups	0	0	1	2	3

	I don't know / I don't remember	No	Yes	NA	Total
Means dependent groups	0	0	1	2	3
Relationship with other constructs	1	12	4	26	43
Time trend	1	13	4	14	32
Total	2	28	17	50	97

Open Science Practices

Note that the following questions were presented once for every construct.

Did you report all the analysis steps in such detail that the reader could reproduce the analyses if they had the data?

- No = 0
- Yes = 1

constructs	responses	missing	distinct	min	median	mean	max
62	54	8	2	0	1	0.93	1

Value	No	Yes
Freq	4	50
Perc	7.4	92.6

Is the syntax or code for all the analysis steps publicly available (e.g., on the Open Science Framework)?

- No = 0
- Yes = 1

constructs	responses	missing	distinct	min	median	mean	max
62	54	8	2	0	1	0.61	1

Value	No	Yes
Freq	21	33
Perc	38.9	61.1

How confident are you that you analyses for the construct (including any assessment of reliability, factor structure and measurement invariance) were ideal for your research design?

- Not at all confident = 1
- Moderately confident = 3

- Extremely confident = 5

constructs	responses	missing	distinct	min	median	mean	max
62	54	8	5	1	3	2.94	5

Value	Extremely confident	Moderately confident	Not at all confident	Slightly confident	Very confident
Freq	5	25	5	11	8
Perc	9.3	46.3	9.3	20.4	14.8
